# SMYD3 promotes aerobic glycolysis in diffuse large B-cell lymphoma via H3K4me3-mediated PKM2 transcription

**DOI:** 10.1038/s41419-022-05208-7

**Published:** 2022-09-03

**Authors:** Tian Tian, Jiwei Li, Di Shi, Yupeng Zeng, Baohua Yu, Xiaoqiu Li, Ping Wei, Xiaoyan Zhou

**Affiliations:** 1 Department of Pathology, Fudan University Shanghai Cancer Center, 270 Dong’an Road, Shanghai, 200032 China; 2 Department of Oncology, Shanghai Medical College, Fudan University, 270 Dong’an Road, Shanghai, 200032 China; 3 Institute of Pathology, Fudan University, 270 Dong’an Road, Shanghai, 200032 China; 4 Cancer Institute, Fudan University Shanghai Cancer Center, 270 Dong’an Road, Shanghai, 200032 China

**Keywords:** Lymphoma, Cancer metabolism

## Abstract

Genetic abnormalities in histone methyltransferases (HMTs) frequently occur in diffuse large B-cell lymphoma (DLBCL) and are related to its progression. SET and MYND domain containing 3 (SMYD3) is an HMT that is upregulated in various tumors and promotes their malignancy. However, to the best of our knowledge, the function of SMYD3 in DLBCL has not been investigated thus far. In the present study, 22 HMT genes related to cancer development were first selected according to current literature, and it was found that high SMYD3 expression was significantly associated with poor progression-free survival in patients with DLBCL. SMYD3 protein levels were upregulated and positively associated with poor prognosis and poor responsiveness to chemotherapy in patients with DLBCL. Functional examinations demonstrated that SMYD3 increased cell proliferation and the flux of aerobic glycolysis in DLBCL cells in vitro and in vivo and decreased cell sensitivity to doxorubicin in vitro. Moreover, SMYD3 could directly bind to specific sequences of Pyruvate Kinase M2 (*PKM2*) and promote DLBCL cell proliferation and aerobic glycolysis via H3K4me3-mediated PKM2 transcription. Clinically, SMYD3 expression positively correlated with that of PKM2, and high SMYD3 was significantly associated with high maximum standardized uptake value (SUVmax) detected by [(18)F]-fluorodeoxyglucose ((18)F-FDG) PET/computed tomography (PET/CT) in DLBCL samples. Concomitant expression of SMYD3 and PKM2 positively correlated with poor progression-free and overall survival in patients with DLBCL and may serve as novel biomarkers in DLBCL.

## Introduction

Diffuse large B-cell lymphoma (DLBCL) is the most common lymphoma that occurs in adults and accounts for approximately one-third of non-Hodgkin’s lymphoma cases worldwide [1]. It is highly aggressive and heterogeneous in clinicopathological features, immunophenotypes, genetic abnormalities, chemotherapeutic response and prognosis [2]. Although the inclusion of rituximab in the cyclophosphamide, hydroxydaunorubicin, oncovin and prednisone (RCHOP)chemotherapy regimen has improved the survival of patients with DLBCL, nearly 40% of patients are resistant to this type of chemotherapy and thus have a poor prognosis [3, 4]. Therefore, it is critical to investigate the molecular mechanisms underlying the progression of DLBCL to identify new molecular biomarkers and therapeutic targets that might improve the prognosis of patients with DLBCL.

Chromatin changes, such as histone methylation occurred in gene promoters, have been linked to carcinogenesis [5]. Histone methyltransferases (HMTs) play crucial roles in epigenetic regulation by controlling histone lysine methylation, which may accelerate cancer progression [6, 7]. A growing body of evidence indicates that dysregulated expression or mutation of various HMTs, such as lysine methyltransferase 2D (KMT2D), CREB-binding protein (CREBBP)and enhancer of zeste 2 polycomb repressive complex 2 subunit (EZH2), are some of the most common gene abnormalities in DLBCL, directly contributing to the molecular pathogenesis and progression of DLBCL [8, 9]. For example, a gain-of-function mutation in EZH2 in DLBCL promotes germinal center proliferation [10]. Recent study has shown that epigenetics-related drugs such as EZH2 inhibitors could improve the responsiveness of patients with DLBCL to chemotherapy [11]. Thus, HMTs might be used as new therapeutic targets in DLBCL. Therefore, the investigation of HMT genes in DLBCL and understanding the underlying epigenetic mechanism in the progression of this disease are crucial to the establishment of therapeutic targets.

SMYD3 is a lysine HMT that functions as a transcriptional potentiator of genes by multiple possible mechanisms including binding to specific DNA sequences (5’-CCCTCC-3’ or 5’-GGAGGG-3’), interacting with transcription factors and trimethylated H3K4 (the fourth lysine of histone 3) tails and so on [12]. Upregulation of SMYD3 has been identified in several types of cancer, such as breast and colorectal cancer, and is associated with tumor proliferation and metastasis [13, 14]. These findings imply that SMYD3 might play a crucial role in tumor progression. However, the role of SMYD3 in DLBCL remains unclear; thus, its function and mechanism of action in the progression of DLBCL should be explored.

Pyruvate kinase M (PKM) 1 and -2 are produced by the alternative splicing of two mutually exclusive exons in the *PKM* gene. PKM1 is expressed in terminally differentiated tissue types, such as the brain and muscle, while PKM2 is expressed in proliferating tissue and tumor cells [15]. The reversion of PKM1 to PKM2 is indispensable for shifting glucose metabolism toward aerobic glycolysis [16]. It has been reported that specific loss of PKM2 suppresses the growth of HCT116 cells [17]. An increased PKM2/PKM1 ratio has been observed in multiple cancer types and is significantly associated with poor overall survival (OS) [18, 19]. PKM2 also acts as a coactivator for transcription factors, implicating in the activation of STAT3, β-catenin, HIF-1α, OCT-4 and ERα signaling pathways in cancer cells to mediate their proliferation and metastasis [15].

In the present study, SMYD3 could promote the proliferation and aerobic glycolysis of DLBCL cells in vitro and in vivo. RNA sequencing (RNA-Seq) analysis indicated that PKM2 was an important target of SMYD3. Further functional assays revealed that SMYD3 promoted DLBCL cell proliferation and glycolysis via H3K4me3-mediated PKM2 transcription. Therefore, SMYD3 and PKM2 may serve as a prognostic panel for DLBCL.

## Results

### SMYD3 upregulation is associated with a poor prognosis in patients with DLBCL

**Fig. 1:**
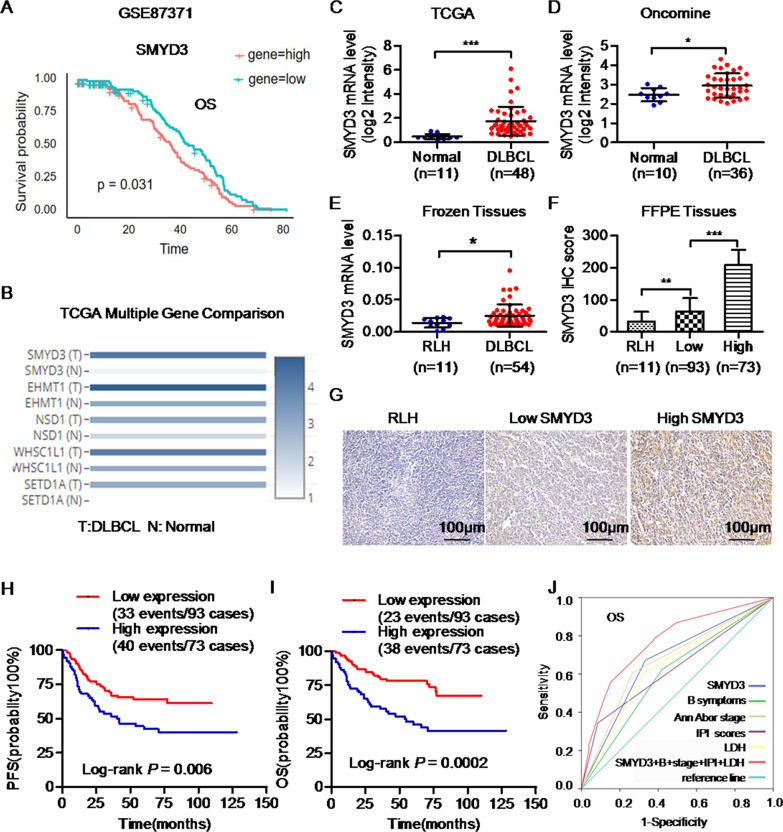
High levels of SMYD3 are associated with a poor prognosis in patients with DLBCL. **A** High mRNA levels of SMYD3 were associated with poor OS in patients with DLBCL from the GSE87371 dataset. **B** The mRNA levels [log_2_(TPM + 1)] of SMYD3, EHMT1, NSD1, WHSC1L1 (NSD3) and SETD1A were significantly higher in DLBCL than those of normal tissue samples in TCGA database. Analysis of (**C**) TCGA and **D** Oncomine databases suggested that the mRNA levels of SMYD3 in DLBCLs were significantly higher than those of normal tissue samples. The **E** mRNA and **F** protein expression levels of SMYD3 in DLBCL clinical samples were significantly higher than those in RLH samples. **G** Immunohistochemical staining images of SMYD3 in RLH samples (H-score, 40) and in DLBCLs with low (H-score, 100) or high (H-score, 260) SMYD3 protein levels. Magnification, x200. Scale bar, 100 μm. Patients of DLBCLs with high SMYD3 protein expression had a shorter PFS (**H**) and a shorter OS (**I**) than those with low SMYD3 protein expression respectively. *P*-values from log-rank tests are shown. **J** ROC curves of OS showing the AUROC of SMYD3 expression combined with Ann Arbor stage, B symptoms, IPI score and serum LDH level vs. AUROCs of each factor alone. DLBCL, diffuse large B-cell lymphoma; SMYD3; SET and MYND domain containing 3; EHMT1, euchromatic histone lysine methyltransferase 1; NSD1/3, nuclear receptor binding SET domain protein 1/3; WHSC1L1, Wolf-Hirschhorn syndrome candidate 1-like 1; SETD1A, SET domain containing 1A histone lysine methyltransferase. ROC Receiver operating characteristic, AUROC Area under the ROC curve, PFS Progression-free survival, OS, Overall survival, TPM Transcripts per million, RLH Reactive lymphoid hyperplasia.

To identify the role of HMTs in DLBCL, 22 HMT genes associated with tumor progression according to previous studies [20–31] (Table S1) were first selected. These genes were then analyzed in multiple DLBCL public datasets. The survival analysis of the GSE87371 dataset using R language revealed that the lower mRNA expression levels of euchromatic histone lysine methyltransferase 1 (EHMT1), nuclear receptor binding SET domain protein (NSD) 1, Wolf-Hirschhorn syndrome candidate 1-like 1(WHSC1L1/NSD3) and SET domain containing 1 A histone lysine methyltransferase (SETD1A) were significantly associated with the poor progression-free survival (PFS) and OS of DLBCL patients (Fig. S1A, B). However, the higher mRNA level of SMYD3 was significantly associated with the poor OS of DLBCL patients in the GSE87371 dataset (Fig. 1A). TCGA database indicated that the mRNA levels of these genes in DLBCL were significantly higher than those of normal tissue samples (Fig. 1B, C; Fig. S1C). The Oncomine database also suggested that the mRNA expression levels of SMYD3 in DLBCL were significantly higher than those of normal tissue samples in the Nature 2009 dataset (Fig. 1D).

The aforementioned results were then validated in clinical samples. The mRNA levels of SMYD3 were significantly upregulated in DLBCL compared with RLH tissue samples (*P* < 0.05; Fig. 1E). Similarly, SMYD3 protein expression was higher in DLBCL than in RLH (*P* = 0.009; Fig. 1F, G).

A total of 166 DLBCL cases were then divided into a high (H-score>120, 73 cases, 44.0%) and a low SMYD3 expression group (H-score < 120, 93 cases, 56.0%), according to the cutoff of the SMYD3 H-score based on the ROC curve. Patients of DLBCL with high levels of SMYD3 protein were more likely to have a higher Ann Arbor stage (III and IV; *P* = 0.018) and a higher level of serum LDH (>240 U/l, *P* = 0.032). Moreover, they exhibited poor responsiveness to chemotherapy (*P* = 0.015), and poor prognosis for this group (*P* = 0.017; Table 1). Kaplan-Meier curves suggested that patients with DLBCL and high SMYD3 expression had poor PFS (*P* = 0.006) and OS (*P* = 0.0002) than patients with low SMYD3 expression (Fig. 1H, I). Univariate analysis indicated that patients with high SMYD3 protein expression levels had a 1.184-fold increase in the risk of disease progression, recurrence or death [hazard ratio (HR) = 2.184; 95% CI, 1.369–3.486; *P* = 0.001; Table 2) and a 2.119-fold increase in the risk of death (HR = 3.119; 95% CI, 1.826–5.328; *P* < 0.001; Table 3). Multivariate analysis confirmed that high SMYD3 protein expression was an independent prognostic marker for poor PFS (HR = 1.728; 95% CI, 1.070–2.791; *P* = 0.025; Table 2) and OS (HR = 2.645; 95% CI, 1.534–4.558; *P* < 0.001; Table 3) in patients with DLBCL. Moreover, the combination of SMYD3 protein expression level with Ann Arbor stage, B symptoms, IPI score and serum LDH level could better predict OS than each factor individually (*P* = 0.0001, the area under the ROC curve (AUROC) = 0.731, Fig. 1J).

**Table 1:** Associations of SMYD3 protein expression with the clinicopathologic characteristics of DLBCLs.

Variables	Total case	Low SMYD3 expression (%)	HighSMYD3 expression (%)	*P*-value
Age				
≤60	104	61 (58.7)	43 (41.3)	0.377
>60	62	32 (51.6)	30 (48.4)	
Sex				
Male	95	52 (54.7)	43 (45.3)	0.699
Female	71	41 (57.7)	30 (42.3)	
Primary site				
Nodal	119	71 (59.7)	48 (40.3)	0.133
Extranodal	47	22 (46.8)	25 (53.2)	
Ann Arbor Stage				
I–II	101	64 (63.4)	37 (36.6)	0.018^*^
III–IV	65	29 (44.6)	36 (55.4)	
B Symptoms				
Yes	82	46 (56.1)	36 (43.9)	0.985
No	84	47 (56.0)	37 (44.0)	
IPI scores				
Low (0–2)	136	81 (59.6)	55 (40.4)	0.051
High (3–5)	30	12 (40.0)	18 (60.0)	
Serum LDH				
Normal (≤ 240)	95	60 (63.2)	35 (36.8)	0.032^*^
High (> 240)	71	33 (46.5)	38 (53.5)	
Type (IHC)				
GCB	81	47 (58.0)	34 (42.0)	0.612
Non-GCB	85	46 (54.1)	39 (45.9)	
Chemotherapy^a^				
Without Rituximab	92	47 (51.1)	45 (48.9)	0.153
With Rituximab	74	46 (62.2)	28 (37.8)	
Chemothrapy response				
CR + PR	86	53 (61.6)	33 (38.4)	0.015^*^
PD + SD	16	4 (25.0)	12 (75.0)	
Relapse or die in 2 years				
Yes	50	21 (42.0)	29 (58.0)	0.017^*^
No	116	72 (62.1)	44 (37.9)	

*DLBCL* Diffuse large B-cell lymphoma, *GCB* Germinal center B cell, *IHC* Immunohistochemistry, *IPI* International Prognostic Index, *LDH* Lactate dehydrogenase, *CR* Complete response, *PR* Partial response, *PD* Progressive disease, *SD* Stable disease.

^a^Regimens without Rituximab include CHOP, CEOP, CTOP, HyperCVAD in our cohort; regimens with Rituximab include R-CHOP,R-CEOP, R-HyperCVAD.

^*^P-values are significant at *P* < 0.05.

**Table 2:** Univariate and multivariate analysis for associations of SMYD3 protein expression with PFS in DLBCLs.

Variables	Univariate analysis			Multivariate analysis		
	HR	95%CI	*P*-value	HR	95%CI	*P*-value
SMYD3						
Low	1 [Reference]			1 [Reference]		
High	2.184	1.369–3.486	0.001^*^	1.728	1.070–2.791	0.025^*^
Age						
≤60	1 [Reference]					
>60	1.016	0.634–1.629	0.947			
Sex						
Male	1 [Reference]					
Female	1.340	0.833–2.155	0.228			
Primary site						
Nodal	1 [Reference]					
Extranodal	1.321	0.810–2.153	0.264			
Ann Arbor Stage						
I–II	1 [Reference]			1 [Reference]		
III–IV	2.725	1.712–4.336	<0.001^*^	1.942	1.186–3.180	0.008^*^
IPI scores						
Low (0–2)	1 [Reference]			1 [Reference]		
High (3–5)	3.661	2.217–6.045	<0.001^*^	1.541	0.807–2.941	0.190
B symptoms						
No	1 [Reference]			1 [Reference]		
Yes	1.912	1.196–3.058	0.007^*^	1.773	1.088–2.887	0.021^*^
Serum LDH						
≤240	1 [Reference]			1 [Reference]		
>240	3.230	2.014–5.083	<0.001^*^	2.365	1.388–4.031	0.002^*^
Type (IHC)						
GCB	1 [Reference]					
Non-GCB	0.743	0.469–1.178	0.207			

*DLBCL* Diffuse large B-cell lymphoma, *GCB* Germinal center B cell, *IHC* Immunohistochemistry, *IPI* International Prognostic Index, *LDH* Lactate dehydrogenase, *CI* Confidence interval, *HR* Hazard’s ratio.

^*^P values are significant at *P* < 0.05.

**Table 3:** Univariate and multivariate analysis for associations of SMYD3 protein expression with OS in DLBCLs.

Variables	Univariate analysis			Multivariate analysis		
	HR	95%CI	*P*-value	HR	95%CI	*P*-value
SMYD3						
Low	1 [Reference]			1 [Reference]		
High	3.119	1.826–5.328	<0.001^*^	2.645	1.534–4.558	<0.001^*^
Age						
≤60	1 [Reference]					
>60	1.007	0.600–1.691	0.978			
Sex						
Male	1 [Reference]					
Female	0.733	0.435–1.237	0.245			
Primary site						
Nodal	1 [Reference]					
Extranodal	1.456	0.859–2.470	0.163			
Ann Arbor Stage						
I-II	1 [Reference]			1 [Reference]		
III-IV	3.157	1.885–5.287	<0.001^*^	1.521	0.809–2.860	0.193
IPI scores						
Low (0–2)	1 [Reference]			1 [Reference]		
High (3–5)	4.484	2.620–7.675	<0.001^*^	2.687	1.466–4.926	0.001^*^
B symptoms						
No	1 [Reference]			1 [Reference]		
Yes	1.993	1.187–3.346	0.009^*^	1.801	1.067–3.040	0.028^*^
Serum LDH						
≤240	1 [Reference]			1 [Reference]		
>240	3.043	1.815–5.102	<0.001^*^	1.740	0.967–3130	0.065
Type (IHC)						
GCB	1 [Reference]					
Non-GCB	0.604	0.363–1.003	0.051			

*DLBCL* Diffuse large B-cell lymphoma, *GCB* Germinal center B cell, *IHC* Immunohistochemistry, *IPI* International Prognostic Index, LDH Lactate dehydrogenase, *CI* Confidence interval, *HR* Hazard’s ratio.

^*^P values are significant at *P* < 0.05.

Together, these results demonstrated that SMYD3 expression was upregulated in DLBCL and that the predictive value of SMYD3 expression levels for poor prognosis was independent of known factors.

### SMYD3 promotes DLBCL cell proliferation in vitro and in vivo

**Fig. 2:**
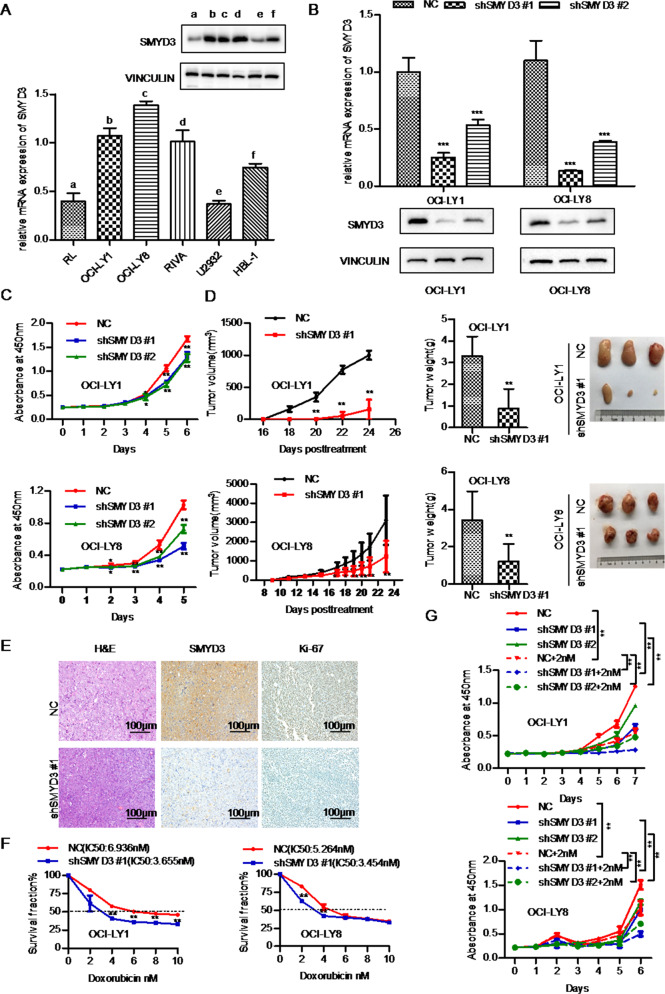
Stable knockdown of SMYD3 suppresses the proliferation of DLBCL cells in vitro and in vivo and increases their sensitivity to doxorubicin in vitro. **A** The mRNA and protein expression levels of SMYD3 in the RL, OCI-LY1, OCI-LY8, RIVA, U2932 and HBL-1 DLBCL cell lines. **B** Knockdown efficiency of SMYD3 mRNA and protein levels in the OCI-LY1 and OCI-LY8 cell lines. **C** Knockdown of SMYD3 expression suppressed DLBCL cell proliferation in the OCI-LY1 and OCI-LY8 cell lines. **D** Tumorigenicity assay of SMYD3-knockdown OCI-LY8 and OCI-LY1 cells. **E** Hematoxylin and eosin and SMYD3 and Ki67 staining of xenograft tissue samples from mice injected with SMYD3-knockdown OCI-LY8 cells. Magnification, x200. Scale bar, 100 μm. **F** IC_50_ values for doxorubicin in OCI-LY1 and OCI-LY8 cell lines were obtained using Cell Counting Kit-8 assays. **G** SMYD3 knockdown cells proliferated much more slowly after treatment with doxorubicin over a period of 6 days in OCI-LY1 and OCI-LY8 cells.^*^P < 0.05, ^**^P < 0.01, ^***^P < 0.001. DLBCL Diffuse large B-cell lymphoma, SMYD3 SET and MYND domain containing 3.

The functional role of SMYD3 in DLBCL was then evaluated. The mRNA and protein levels of SMYD3 were examined in six DLBCL cell lines. The OCI-LY1 and OCI-LY8 cell lines, which had relatively high SMYD3 mRNA and protein expression levels, were selected for SMYD3 stable knockdown using shRNA (Fig. 2A). Stable transfection of SMYD3 shRNA efficiently silenced the mRNA and protein expression of SMYD3 in OCI-LY1 and OCI-LY8 cells (Fig. 2B). In addition, CCK-8 assays performed over a period of 5 days showed that OCI-LY1 and OCI-LY8 cells proliferated much more slowly following knockdown of SMYD3 with two independent shRNA constructs (Fig. 2C). Stable overexpression of SMYD3 was also carried out in the HBL1 cell line. A CCK-8 assay revealed that HBL1 cells proliferated slightly more rapidly following SMYD3 overexpression, compared with vector control cells (Fig. S2), as SMYD3 was innately expressed at high levels in these cells.

To further validate the effect of SMYD3 on proliferation in vivo, SMYD3-knockdown OCI-LY1 and OCI-LY8 cells and respective control cells were subcutaneously injected into nude mice. The volumes, proliferation rate and weight of the tumors formed by the SMYD3-knockdown OCI-LY1 and OCI-LY8 cells were significantly decreased compared with the respective control cells (Fig. 2D). IHC staining also showed that Ki-67 protein expression decreased in SMYD3-knockdown OCI-LY8 xenografts (Fig. 2E).

The role of SMYD3 in responsiveness to doxorubicin treatment was then assessed. CCK-8 assays revealed that SMYD3 knockdown significantly decreased the IC_50_ value of doxorubicin in OCI-LY1 and OCI-LY8 cell lines (Fig. 2F), and SMYD3 knockdown cells proliferated much more slowly after treatment with doxorubicin over a period of 6 days (Fig. 2G). These results indicated that SMYD3 knockdown increased DLBCL cell susceptibility to doxorubicin. In addition, the role of SMYD3 in responsiveness to vincristine was also examined. CCK-8 assay showed that the IC_50_ value of vincristine was significantly decreased after SMYD3 knockdown and SMYD3 knockdown cells proliferated much more slowly after treatment with vincristine in OCI-LY1 and OCI-LY8 cell lines (Fig S5).

Together, these results suggested that SMYD3 played an important role in promoting DLBCL cell proliferation in vitro and in vivo and that SMYD3 induced doxorubicin and vincristine resistance in DLBCL cells in vitro.

### SMYD3 enhances glycolysis in DLBCL cells in vitro and in vivo

To further explore the functional role of SMYD3 in DLBCL, RNA-Seq was carried out on total RNA isolated from stable SMYD3-knockdown OCI-LY8 cells transfected with two independent shRNA (shSMYD3 #1 and shSMYD3 #2) and the negative control (NC)OCI-LY8cells. RNA-Seq identified 11738 differentially expressed genes. With a fold-change cutoff of 1.5 or 0.7 and a *P*-value cutoff of 0.05, gene expression profiling revealed a total of 2,267 overlapping differentially expressed genes in shSMYD3 #1 and shSMYD3 #2 versus (vs). NC (Fig. S3A). We then performed GSEA analysis of all the 11738 differentially expressed genes of shSMYD3 #1 vs NC and shSMYD3 #2 vs NC, respectively. We focused on the overlapping enriched pathways of those two groups. GSEA of KEGG enrichment showed that the ‘Glycolysis-Gluconeogenesis’ pathway was upregulated in phenotype shSMYD3 #1 (normalized enrichment score (NES) = 1.448, normalized *P* = 0.040, false discovery rate (FDR) *q* = 0.134) and shSMYD3 #2 (NES = 1.762, normalized *P* = 0.005, FDR q = 0.078; Fig. S3B, C).

**Fig. 3:**
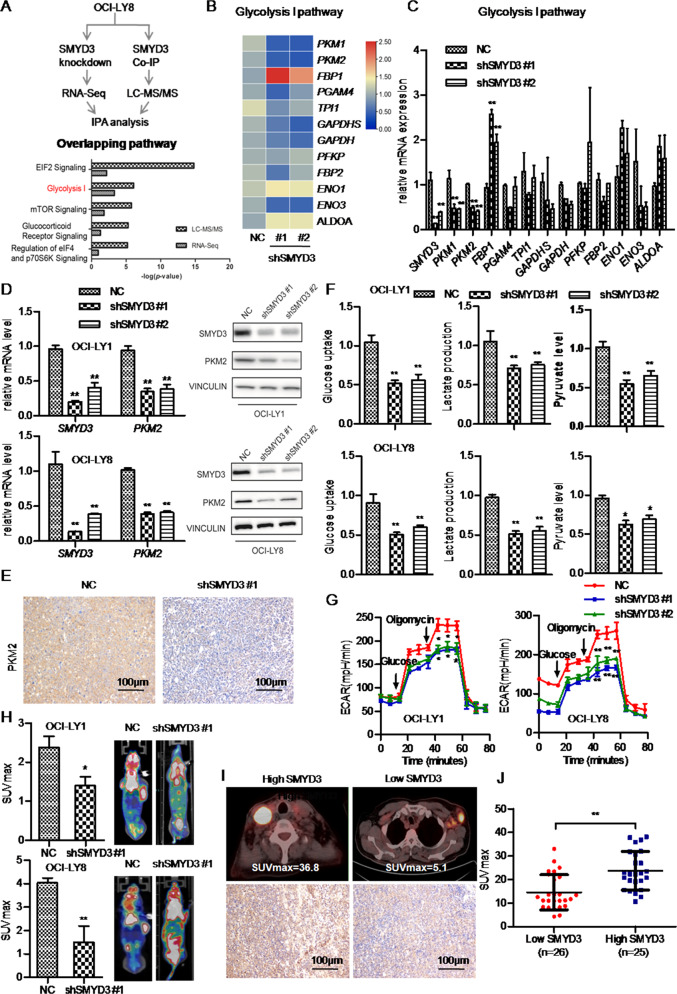
SMYD3 promotes PKM2 expression and aerobic glycolysis in DLBCL cell lines in vitro and in vivo. **A** Flowchart of IPA analysis and the top 5 significant pathways of DEGs in integrated analysis of LC-MS/MS and RNA-Seq data. **B**, **C** The mRNA levels of DEGs enriched in ‘glycolysis’ were validated using reverse transcription-quantitative PCR in SMYD3-knockdown OCI-LY8 cells and NC cells and were visualized in the heatmap. (The names of the different columns in the heatmap were shown). **D** The mRNA and protein expression of PKM2 decreased following SMYD3knockdown in the OCI-LY1 and OCI-LY8 cell lines. **E** SMYD3 and PKM2 staining in xenograft tissue samples from mice injected with SMYD3-knockdown OCI-LY8 and NC cells. Magnification, x200. Scale bar,100 μm. **F** Glucose uptake, cellular pyruvate levels, lactate production and **G** ECAR significantly decreased following SMYD3 knockdown in the OCI-LY1 and OCI-LY8 cell lines. **H** SMYD3 knockdown inhibited the glucose uptake of DLBCLs in vivo. **I** Illustration of SUVmax from PET-CT scans of patients with DLBCL and high or low SMYD3 protein expression. **J** High SMYD3 protein expression was significantly associated with the SUVmax of patients with DLBCL. ^*^P < 0.05, ^**^P < 0.01, ^***^P < 0.001. DLBCL Diffuse large B-cell lymphoma, SMYD3 SET and MYND domain containing 3, PKM2 Pyruvate kinase M2, DEG Differentially expressed gene, IPA Ingenuity pathway analysis, LC Liquid chromatography, MS Mass spectrometry, RNA-Seq RNA sequencing, NC Negative control, PET Positron emission tomography, CT Computed tomography, ECAR Extracellular acidification rate, SUVmax Maximum standardized uptake value.

Next, we investigated the proteins physically interacted with SMYD3 and explored the function played by SMYD3 in binding with other factors. An endogenous Co-IP assay using an anti-SMYD3 antibody was carried out in OCI-LY8 cells. The proteins co-purified with SMYD3were resolved by SDS-PAGE, and major protein bands were excised and subjected to LC-MS/MS analysis. It identified 541 differentially expressed proteins which were quantifiable (Table S2, SMYD3.vs.IgG_quant) and among them 42 proteins were significantly differentially expressed (significance ≥ 20, Fold Change ≥ 1.5, unique peptides ≥ 2, Table S2, SMYD3.vs.IgG_regulated). Then IPA of these differentially expressed proteins was performed to explore the enriched pathways. Meanwhile, the differentially expressed genes examined by RNA-seq were also analyzed by IPA to enrich the associated pathways. Then we focused on the overlapping pathways between LC-MS/MS and RNA-seq (Table S3). It revealed that the ‘glycolysis I’ was one of the top 5 overlapping signaling pathways (Fig. 3A). Then we performed RT-qPCR assay to confirm the mRNA expression level of genes enriched in the ‘glycolysis I’ pathway. The results showed that Pyruvate Kinase M1 (*PKM1*) and Pyruvate Kinase M2 (*PKM2*) significantly decreased (*P* < 0.01) and Fructose-Bisphosphatase 1 (*FBP1*) significantly increased (*P* < 0.01) following SMYD3 knockdown (Fig. 3B, C). However, Mann-Whitney test showed that Phosphoglycerate Mutase Family Member 4 (*PGAM4*), Triosephosphate Isomerase 1 (*TPI1*), Glyceraldehyde-3-Phosphate Dehydrogenase, Spermatogenic (*GAPDHS*), Glyceraldehyde-3-Phosphate Dehydrogenase (*GAPDH*), Phosphofructokinase, Platelet (*PFKP*), Fructose-Bisphosphatase 2 (*FBP2*), Enolase 1(*ENO1*), Enolase 3 (*ENO3*), and Aldolase, Fructose-Bisphosphate A (*ALDOA*) did not significantly change after SMYD3 knockdown (Fig. 3B, C). IHC staining revealed that the staining intensity and the staining proportion of PKM1 were markedly low (H-score: 0) and that the PKM2 staining intensity was moderate and the staining proportion of PKM2 was about 70% (H-score: 140) in the DLBCL FFPE sample (Fig. S4). Therefore, PKM2 was further examined in subsequent experiments. RT-qPCR and western blot analysis demonstrated that the mRNA and protein levels of PKM2 were significantly downregulated after SMYD3 knockdown in OCI-LY1 and OCI-LY8 cells (Fig. 3D). In addition, IHC staining revealed that low SMYD3 expression in xenograft tumor tissue was associated with weak PKM2 staining (Fig. 3E).

The next experiments were carried out to determine whether SMYD3 modulated glycolysis in DLBCL. SMYD3 knockdown significantly reduced glucose uptake, lactate production and pyruvate level in OCI-LY1 and OCI-LY8 cells (Fig. 3F) and decreased the ECAR (Fig. 3G), which reflected glycolytic flux. To validate the effects of SMYD3 on glycolysis in vivo, PET/CT scan was used to measure glucose uptake in tumor xenografts from nude mice. The SMYD3 knockdown DLBCL tumors showed a lower SUVmax value compared with the NC, suggesting decreased glucose uptake (Fig. 3H). The association between SMYD3 expression and the SUVmax was further analyzed in clinical DLBCL samples. DLBCLs with the SUVmax value before therapy according to PET-CT were chosen (*n* = 51). The Mann-Whitney U test showed that the SUVmax value of the high SMYD3 expression group was significantly higher than that of the low SMYD3 expression group (*P* = 0.0001, Fig. 3I, J).

These results indicated that SMYD3 upregulated the mRNA and protein expression of PKM2 and promoted glycolysis in DLBCL in vitro and in vivo. The association between SMYD3 expression with glucose uptake was also validated in clinical samples and it demonstrated that higher SMYD3 expression was associated with higher SUVmax value in DLBCL.

### SMYD3 promotes PKM2 expression via trimethylation of H3K4 in DLBCL

**Fig. 4:**
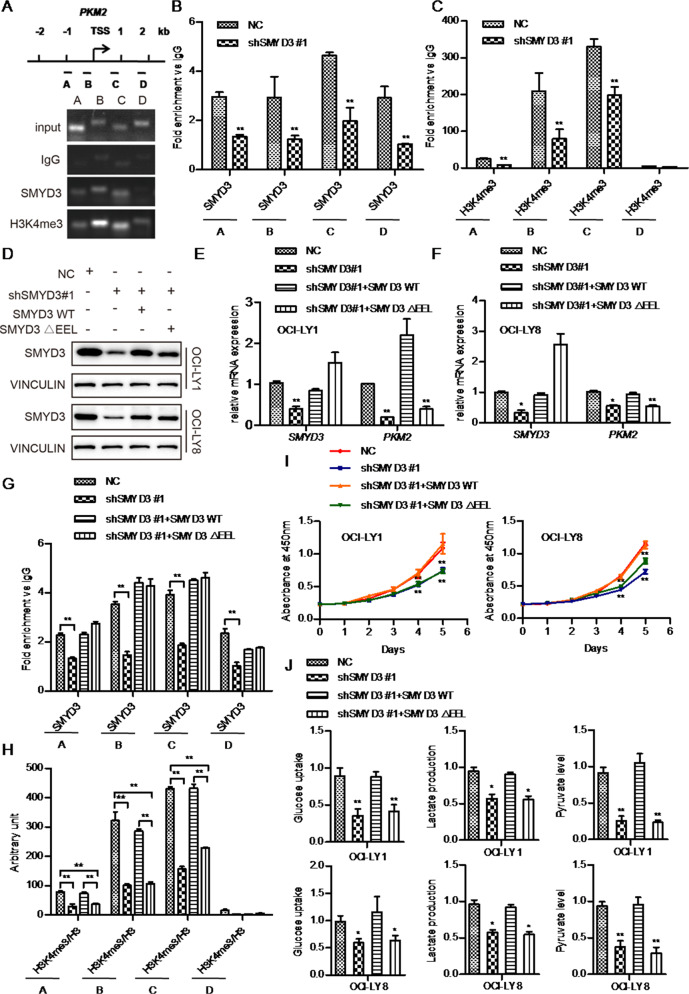
SMYD3 promotes PKM2 transcription and the malignant phenotype of DLBCL via H3K4me3. **A** DNA agarose gel electrophoresis following ChIP-PCR showing the binding region of SMYD3 2000 bp upstream/downstream of the TSS of *PKM2*.ChIP-qPCR assays showing the enrichment of SMYD3 (**B**) and H3K4me3 (**C**) at potential binding sites A, B, C and D of *PKM2*. **D** Protein expression levels of SMYD3 following overexpression of SMYD3 WT and ΔEEL in SMYD3-knockdown DLBCL cell lines. **E**, **F** PKM2 mRNA levels after overexpression of SMYD3 WT and ΔEEL in SMYD3-knockdown DLBCL cell lines. **G**, **H** The SMYD3 and H3K4me3 enrichment levels in the *PKM2* regulatory regions after overexpression of SMYD3 WT and ΔEEL in SMYD3-knockdown OCI-LY8 cells. **I** SMYD3-knockdown OCI-LY1 and OCI-LY8 cells were transfected with SMYD3 WT or SMYD3 ΔEEL, and the proliferation rates were measured using Cell Counting Kit-8 assays. **J** Changes in glucose uptake, lactate production and pyruvate levels. ^*^P < 0.05, ^**^P < 0.01, ^***^P < 0.001. DLBCL Diffuse large B-cell lymphoma, SMYD3 SET and MYND domain containing 3, PKM2 Pyruvate kinase M2, TSS Transcription start site, ChIP Chromatin immunoprecipitation, qPCR Quantitative PCR, WT Wild-type.

It has been previously reported that SMYD3 binds to a specific DNA sequence (5’-CCCTCC-3’ or 5’-GGAGGG-3’) in its target genes to regulate gene transcription [32]. Therefore, we searched for this specific SMYD3 binding sequence around the transcription start site (TSS) of *PKM2. PKM2* contained potential SMYD3 binding elements within a region 2000 bp upstream or downstream of the TSS. Thus, it was hypothesized that SMYD3 might directly regulate the transcription of *PKM2*. To test this possibility, a ChIP assay was performed to determine whether SMYD3 could bind to *PKM2*. ChIP-qPCR and subsequent DNA gel electrophoresis showed that SMYD3 could bind to four specific sites (A(−1062 to −946bp), B(−282 to −129 bp), C(860 to 978 bp), D(1793 to 1980 bp), Fig. 4A) within a region 2000 bp upstream or downstream of the TSS of *PKM2* and high levels of SMYD3 occupancy at these sites in the negative control OCI-LY8 cells (NC cells). Compared with the NC cells, SMYD3 enrichment was markedly decreased following SMYD3 knockdown (Fig. 4B). In addition, SMYD3 occupancy was higher at the proximal promoter binding sites (regions B and C) than at regions A and D (Fig. 4A, B). This result supported the notion that SMYD3 takes part in the early steps of gene transcription and facilitates the transition from transcription initiation to early elongation [33].

It has been demonstrated that SMYD3 mediates the trimethylation of H3K4 [32, 33]. Therefore, the next experiments aimed to determine whether SMYD3 regulated *PKM2* transcription by trimethylating H3K4. ChIP-qPCR assays showed that H3K4me3 enrichment at the binding sites was significantly reduced following SMYD3 knockdown in OCI-LY8 cells compared with NC cells (Fig. 4C). Similarly, the H3K4me3 enrichment was higher at the regions B and C than at regions A and D. These results indicated that SMYD3 played a major role in upregulating H3K4me3 levels on *PKM2*.

It was demonstrated that the conserved amino-acid sequence (GEELXXXY) in the SET domain of SMYD3 was responsible for the interaction with SAM and the HMTase activity [33]. Therefore, the mutant protein (SMYD3 ΔEEL) that lacked the EEL motif did not interact with 3H-labelled SAM and thus was methylation defective [33]. To further confirm that SMYD3 promoted *PKM2* transcription via H3K4me3, rescue assays in SMYD3-knockdown OCI-LY1 and OCI-LY8 cells were carried out by transfecting wild-type SMYD3 (SMYD3 WT) or a methylation defective mutant (SMYD3ΔEEL) (Fig. 4D). RT-qPCR assay showed that shSMYD3 cells reconstituted with SMYD3ΔEEL failed to rescue PKM2 mRNA levels in OCI-LY1 and OCI-LY8 cells (Fig. 4E, F). The rescue ChIP assay revealed that the enrichment level of SMYD3 in the PKM2 regulatory regions in the SMYD3-WT and SMYD3-Delta EEL cells almost restored to the level of NC cell lines and there were no significant differences between SMYD3-Delta EEL and SMYD3-WT (Fig. 4G). However, the enrichment level of H3K4me3 was significantly decreased in SMYD3-Delta EEL cells compared with the NC cells and the SMYD3-WT cells respectively (*P* < 0.01, Fig. 4H). In the meanwhile, SMYD3 WT fully restored PKM2 mRNA levels (Fig. 4E, F) and H3K4me3 enrichment compared with NC cells (Fig. 4G, H). CCK-8 and metabolic assays also revealed that the transfection with SMYD3 WT, but not SMYD3ΔEEL, restored cell viability, glucose uptake, pyruvate levels and lactate production in OCI-LY1 and OCI-LY8 cells (Fig. 4I, J). These findings suggested that the HMT activity was required to promote SMYD3- mediated transcriptional activation on the *PKM2* gene and to promote the proliferation and glycolysis of DLBCL cells mediated by SMYD3.

### SMYD3 protein expression levels were positively associated with PKM2 protein expression and poor responsiveness to chemotherapy in DLBCL

**Fig. 5:**
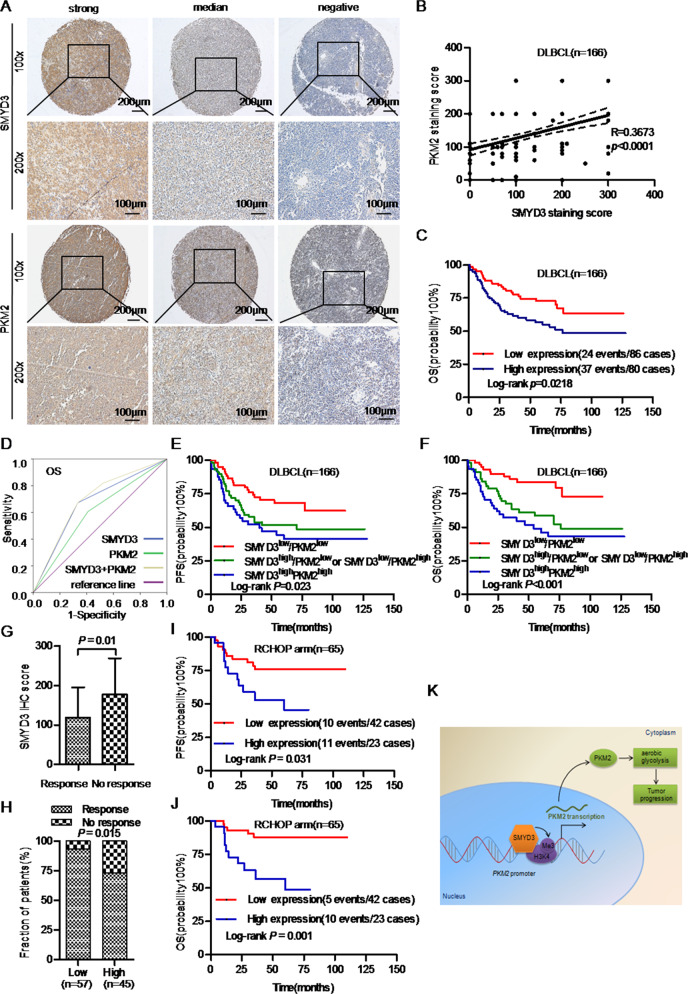
Associations of SMYD3 protein expression with PKM2 expression and the responsiveness to chemotherapy in DLBCL. **A** Representative images of SMYD3 and PKM2 immunohistochemical staining in DLBCL tissue sample were acquired using X10 and X20 objectives. Scale bars, 200 μm and 100 μm. **B** SMYD3 protein expression was positively associated with that of PKM2 in patients with DLBCL (*R* = 0.4712; *P* = 0.0002). **C** Kaplan-Meier survival curves showing that high PKM2 protein expression was associated with poor OS in patients with DLBCL. **D** ROC curves of the combination ofSMYD3 and PKM2 protein expression. Patients in the SMYD3^high^/PKM2^high^ group experienced poor (**E**) PFS and **F** OS, while those in the SMYD3^low^/PKM2^low^ group experienced good PFS and OS. **G** The SMYD3 IHC score of the response group was significantly lower than that of the no-response group. **H** Patients with high SMYD3 expression had a lower response rate than those with low SMYD3 expression. Patients with high SMYD3 expression had poor (**I**) PFS and **J** OS following RCHOP therapy. **K** A schematic model of the mechanism underlying the role of SMYD3 in DLBCL aerobic glycolysis through regulating PKM2 transcription via H3K4me3. DLBCL Diffuse large B-cell lymphoma, SMYD3 SET and MYND domain containing 3, PKM2 Pyruvate kinase M2, ROC Receiver operating characteristic, PFS Progression-free survival, OS Overall survival, H3K4me3 Trimethylated histone 3 lysine 4, RCHOP Rituximab,cyclophosphamide, hydroxydaunorubicin, oncovin and prednisone.

To confirm the relationship between SMYD3 and PKM2, the expression levels of PKM2 were analyzed in clinical samples. IHC staining showed that DLBCL with high SMYD3 protein expression had strong PKM2 staining (Fig. 5A). Spearman’s correlation analysis showed that PKM2 protein expression in 166 DLBCL cases was positively associated with that of SMYD3 (R = 0.3673, *P* < 0.0001; Fig. 5B). Patients with DLBCL and high PKM2 protein expression levels were more likely to relapse or die within two years (*P* = 0.046; Table S4). Kaplan-Meier curves showed that high PKM2 protein expression was significantly associated with poor OS (*P* = 0.0218; Fig. 5C). Multivariate Cox analysis confirmed that PKM2 protein expression was significantly associated with OS despite other clinicopathological parameters (HR = 2.055; 95% CI, 1.217–3.471; *P* = 0.007; Table S5). Moreover, ROC curves showed that the combination of SMYD3 and PKM2 protein expression levels could better predict OS than each protein individually (*P* = 0.0001, AUROC = 0.690, Fig. 5D). Furthermore, DLBCLs with high expression of both SMYD3 and PKM2 were associated with poor PFS (*P* = 0.023) and OS (*P* < 0.001; Fig. 5E, F).

The association between SMYD3 protein expression and responsiveness to chemotherapy in patients with DLBCL was also evaluated. The Mann-Whitney U test showed that the SMYD3 IHC score of the response group (CR and PR) was significantly lower than that of the no-response group (PD and SD) (*P* = 0.01; Fig. 5G). Moreover, patients with high SMYD3 expression had a lower response rate than those with low SMYD3 expression (*P* = 0.015, Fig.5H). Univariate logistic regression analysis showed that high SMYD3 expression was associated with a poor response (PD and SD) to chemotherapy in DLBCL [odds ratio (OR) = 4.818; 95% CI, 1.434–16.193; *P* = 0.011; Table S6)]. Multivariate regression analysis revealed that high SMYD3 expression was an independent predictive factor for PD and SD to chemotherapy (OR = 4.108; 95% CI, 1.108–15.221; *P* = 0.035, Table S6). Moreover, in cases with RCHOP treatment (*n* = 65) from cohort 1, patients with high SMYD3 expression had poorer PFS and OS (Fig. 5I, J), which suggested that high SMYD3 expression was associated with poor responsiveness to this treatment regimen.

Thus, SMYD3 and PKM2 might serve as a prognostic panel for DLBCL. In addition, SMYD3 was an independent predictive biomarker for poor responsiveness to chemotherapy in DLBCL.

## Discussion

The results in the present study demonstrated that SMYD3 was markedly upregulated and high SMYD3 expression was positively associated with poor prognosis and poor responsiveness to chemotherapy in DLBCL. Knockdown of SMYD3 inhibited the proliferation and aerobic glycolysis of DLBCL cells in vitro and in vivo. It was also demonstrated that SMYD3 knockdown increased the sensitivity of DLBCL cells to doxorubicin and vincristine in vitro. Mechanistically, SMYD3 directly bound to the promoter region of *PKM2* and promoted *PKM2* transcription by trimethylating H3K4.

SMYD3 is upregulated in various types of tumors, including colorectal, lung and breast cancer, and is associated with a poor prognosis [34, 35]. Consistent with these findings, our bioinformatics analysis results and clinical validation demonstrated that SMYD3 expression levels were upregulated in DLBCL and positively correlated with a poor prognosis in DLBCL. However, our survival analysis of GEO data showed that the lower expression of EHMT1, NSD1, NSD3, SETD1A was positively associated with the poor PFS and OS in DLBCLs. The discrepancies between HMTs might be caused by the substrates and the degree of methylation (mono-me1/di-me2/tri-me3) and by various proteins that interact with HMTs which affect their selection of residue to modify or the degree of methylation at a specific site [36, 37].

Our study also demonstrated that knockdown of SMYD3 increased cell sensitivity to doxorubicin and vincristine in DLBCL cell lines and high expression of SMYD3 in DLBCL FFPE tissues was associated with poor response to the chemotherapy. Doxorubicin and vincristine were two different types of cytotoxic drugs via different mechanisms of action and were the major drugs of RCHOP chemotherapy in DLBCL. These results suggested that SMYD3 might induce the multi-drug resistance (MDR) phenotype of DLBCL cells. MDR described a phenomenon that lead to resistance to multiple drugs with completely different structure and mechanism of actions [38]. Based on the results presented in this paper, we speculated that SMYD3 might induce MDR phenotype by promoting the glycolysis of DLBCL cells, which provided more ATP for drug efflux transporters and increased acidification of the tumor microenvironment in favor of the catalytic activity of the efflux transporter [38, 39]. Besides, SMYD3 might induce MDR by promoting PKM2 expression, as PKM2 was reported to increase the expression of the efflux transporter Pgp/ABCB1 [40]. The function of SMYD3 in inducing the MDR phenotype in DLBCL and the underlying mechanisms needed to be elucidated in the future researches.

Our RNA-Seq data and GSEA analysis revealed that gene set of ‘Glycolysis-Gluconeogenesis’ was upregulated after SMYD3 knockdown. IPA analysis of LC-MS/MS and RNA-Seq data showed that ‘Glycolysis’ pathway was one of the top overlapping pathways, which indicated that SMYD3 might regulate glycolysis both in protein interaction and transcriptional regulation level. Our following RT-qPCR assay demonstrated that SMYD3 knockdown significantly upregulated the mRNA expression of *FBP1* and downregulated the mRNA level of *PKM2*, but the other enriched genes (*PGAM4, TPI1, PFKP, ENO1, ALDOA*)was not significantly changed. Gluconeogenesis was the opposite process of glycolysis, resulting in the generation of glucose. FBP1 was one of the rate-limiting enzymes in gluconeogenesis, catalyzing fructose 1,6-bisphosphate to fructose 6-phosphate. It was reported that FBP1 antagonized glycolysis and thus inhibited tumor proliferation in many types of tumors [41–43]. Hidenari et al. [44] demonstrated that ectopic FBP1 overexpression inhibited tumor growth and intracellular glucose uptake by reducing aerobic glycolysis in hepatocellular carcinoma cells (HCCs), and also found that KEGG- Glycolysis-Gluconeogenesis was enriched in HCCs expressing high levels of FBP1, which indicated the normal glucose metabolism. Therefore, we assumed that SMYD3 might inhibit gluconeogenesis and thus promote glycolysis by downregulating FBP1 expression. The underlying mechanism of SMYD3 in regulating FBP1 and its significance in DLBCL metabolism and progression needed to be investigated in the future study.

PKM1 and PKM2 are two isoforms of PKM. PKM1 is tetrameric and has higher constitutive activity, while PKM2 acts as a monomer or dimer modulated by allosteric regulation [16]. The present study demonstrated that SMYD3 inhibited the transcription of PKM2. The protein expression of PKM1 was very low in DLBCL tissue. Recent study has reported that the expression of PKM1 tends to be cancer-specific and may have tumor-suppressive and tumor-promoting roles based on varying contexts [17]. Therefore, it is crucial to further investigate the expression and the role of PKM1 in DLBCL cells. To date, few investigators have explored the function of SMYD3 in regulating the transcription of glycolysis-related genes in tumor cells, which was the novel aspect of our study.

It has been reported that SMYD3 promoted tumor proliferation and metastasis by potentiating specific genes transcription in many cancers [12–14]. The underlying mechanism was that SMYD3 could bind with transcriptional cofactors and was recruited to the target genes through multiple mechanisms including interaction with specific DNA sequence, transcription factors and H3K4Me3-modifed promoter-proximal nucleosomes [12]. Sarris et al. [12] demonstrated that SMYD3 was recruited to the promoters of target genes by interacting with RNA Pol-II and trimethylated H3K4 tails to potentiate target genes transcription in DEN-induced liver cancer. Kim et al. demonstrated [32] that SMYD3 could bind to specific DNA sequences and interact directly with PC4 cooperating to promote proliferation genes transcription in colon cancer cells.

The current study has not demonstrated the factors interacting with SMYD3 in gene transcriptional regulation. However, our LC-MS/MS assay has identified several significant SMYD3-interacting proteins, such as Activating Transcription Factor 7 Interacting Protein (ATF7IP), Histone H3.3B and so on. The proteins interacted with SMYD3 and their significance in gene transcriptional regulation in DLBCL needed to be elucidated in our future study.

Sarris et al. [12] observed a bimodal density profile of SMYD3 enrichment at the promoters, two double peaks at the two sides of TSS. In line with this result, our ChIP assay showed that SMYD3 enrichment at the promoter of PKM2 was more pronounced at the left and right sides of the TSS. However, their unbiased search did not detect the previously described SMYD3 binding site “CCTCCC”. Kim et al. demonstrated that SMYD3 promoted gene transcription by binding to “CCCTCC” or “GGAGGG” via H3K4me3 and by interacting with PC4 in bladder and colon cancer cells [32]. In our study, ChIP assay confirmed that SMYD3 bound to “CCCTCC” or “GGAGGG” at the two sides of the TSS of *PKM2* in DLBCL cells. We speculated that interacting with specific DNA sequences might be one of the mechanisms of SMYD3 in regulating *PKM2* transcription in DLBCL cells. Whether SMYD3 was recruited the promoter of *PKM2* by binding with transcription factors or trimethylated H3K4 tails in DLBCL needed to be elucidated in the future study.

Our ChIP-qPCR assays showed that SMYD3-knockdown OCI-LY8 cells displayed a decrease of H3K4me3 at the promoter region of *PKM2*. Rescue assays demonstrated that the HMT activity was required to promote SMYD3-mediated transcriptional activation on the *PKM2* gene. However, our present experiment was not sufficient to demonstrate that SMYD3 directly methylated H3K4 at the promoter. The study by Kim et al. demonstrated that SMYD3 could directly trimethylated H3K4 in vitro and its HMT activity was necessary for the gene activation and tumor promoting function of SMYD3 in colon cancer cells [32]. However, some studies have shown that SMYD3-mediated genes activation was independent on its methylation activity. Fenizia et al. demonstrated that SMYD3 promoted the transcription of EMT genes, independently of SMYD3 methylation activity [14]. We speculated the discrepancy might be caused by the tumor heterogeneity and by regulating different genes. Whether SMYD3 directly methylated H3K4 in DLBCL cells needed to be investigated in the future study.

SMYD3 also methylated other histones substrates such as H4K5 and H4K20. Van Aller et al. [45] demonstrated that SMYD3 catalyzed histone H4 monomethylation at lysine 5 (H4K5me). However, the molecular functions of H4K5 methylation in SMYD3-mediated oncogenic phenotypes remain unclear. Vieira et al. [46] reported that SMYD3 suppressed the expression of cyclin D2 through H4K20me3 and contributed to a more aggressive phenotype of prostate cancer. Therefore, further investigation is needed to investigate other substrates of SMYD3 and their function in DLBCL.

In summary, the current study revealed a new positive regulator of aerobic glycolysis, SMYD3, which is significantly overexpressed in DLBCL tissue and is positively associated with Ann Arbor stage, serum LDH level and poor prognosis and poor response to chemotherapy in patients with DLBCL. SMYD3 promoted DLBCL cell proliferation and aerobic glycolysis by promoting PKM2 transcription via H3K4me3 (Fig. 5K). These observations elucidate the role of the SMYD3/H3K4me3/PKM2 axis in DLBCL and indicate that SMYD3 may be a novel prognostic biomarker and/or therapeutic target for DLBCL.

## Materials and methods

### Patients and tissue samples

Two cohorts consisting of 217 primary DLBCL tissue samples were collected from patients treated at Fudan University Shanghai Cancer Center (FUSCC). All cases were diagnosed by two experienced pathologists (Yu and Li) independently in a blind manner according to the criteria of the World Health Organization classification of hematopoietic and lymphoid tissue. The inclusion criteria included available complete clinicopathological data, including age, sex, International Prognostic Index (IPI) scores, Ann Arbor stage, B symptoms, serum lactate dehydrogenase (LDH), and molecular type identified by immunohistochemistry (germinal center B-cell like (GCB) versus non-GCB), no preoperative anticancer treatment and available chemotherapy response data and survival data (more than one year of follow-up). Cohort 1 included 166 cases of DLBCLs and 11 reactive lymphoid hyperplasias (RLHs) between 2005 and 2011. A total of 102 cases of DLBCLs had been evaluated as complete response (CR, 50 cases), partial response (PR, 36 cases), progressive disease (PD, 9cases) and stable disease (SD, 9 cases) by clinical evidence and PET/CT after chemotherapy. We identified cases of CR and PR as the response group and cases of PD and SD as the no response group. We collected the formalin-fixed, paraffin-embedded (FFPE) tissue blocks of 166 DLBCLs and 11 RLHs to construct tissue microarrays for immunohistochemical analysis, and 54 DLBCL and 11 RLH frozen tissues were collected for reverse transcription-quantitative PCR (RT-qPCR)analysis. Cohort 2 included 51 cases of DLBCLs between 2009 and 2013 that had available SUVmax data detected by PET/CT and 51 DLBCL FFPE blocks were collected to construct tissue microarrays.

In addition, we downloaded datasets from GSE87371 (https://www.ncbi.nlm.nih.gov/geo/), The Cancer Genome Atlas (TCGA) (http://cancergenome.nih.gov/), and Oncomine (https://www.oncomine.org/resource/main.html) to perform bioinformatics analysis to explore gene expression and its association with prognosis.

### RNA isolation and RT-qPCR

RNA was extracted from fresh frozen tissue samples and cell lines using Trizol®reagent (Invitrogen; Thermo Fisher Scientific, Inc.). Total RNA was then reverse transcribed to cDNA using the Prime Script RT Reagent Kit (Takara Biotechnology Co., Ltd.) according to the manufacturer’s protocol. The resulting cDNA samples were subjected to qPCR amplification with an ABI 7900HT qPCR instrument (Applied Biosystems) using SYBR Green (Takara Biotechnology Co., Ltd) and primers for detection of the relative SMYD3, PKM1, PKM2 mRNA levels and β-actin, which was used as an internal control. The primers used for qPCR were listed in Table S7. The data were calculated using the 2^-ΔΔCT^ relative quantification method. The qRT-PCR assay was performed in triplicate and repeated independently three times.

### Tissue microarray and immunohistochemistry

DLBCL tissue microarray (TMA) blocks were constructed using 166 blocks of primary DLBCL and 11 RLH FFPE tissues from cohort 1 and 51 DLBCL FFPE tissues from cohort 2. IHC of SMYD3, PKM2, and Ki-67 expression was performed on 4-μm-thick TMA sections using anti-SMYD3 (Abcam, ab187149, 1:400 dilution), anti-Ki-67 (Abcam, ab92742, 1:500 dilution) and anti-PKM2 (Cell Signaling Technology, #4053, 1:400 dilution) antibodies. The H scoring system (ranging between 0 and 300) was used to semiquantitatively score the expression levels of SMYD3 and PKM2 by two experienced pathologists (Yu and Li) based on the staining intensity and proportion of positively stained cells. We then used the cutoff value calculated by the receiver operating characteristic (ROC) curve to divide patients into groups of high vs. low expression of SMYD3 and PKM2.

### Cell lines and culture

The human DLBCL cell lines OCI-LY1 and OCI-LY8 were kindly provided by Dr B. Hilda Ye (Albert Einstein College of Medicine, USA) and RL, RIVA, U2932, HBL1 cell lines were kindly given by Dr Zebing Liu (Renji Hospital, Shanghai Jiaotong University School of Medicine, Shanghai, China). The 293 T cell line was purchased from the American Type Culture Collection (ATCC) (Manassas, VA). The OCI-LY1 and OCI-LY8 cell lines were maintained in Iscove’s Modified Dulbecco’s Medium (IMDM) (Gibco, BRL Co., Ltd.,USA). The RL, RIVA, U2932, HBL1 cell lines were maintained in RPMI-1640 (Gibco, BRL Co., Ltd.,USA). 293 T cells were cultured in DMEM medium. All cells were supplemented with 10% fetal bovine serum (FBS) (Gibco, BRL Co., Ltd.,USA), 100 U/mL penicillin and 100 μg/mL streptomycin (Gibco, BRL Co., Ltd.,USA). Cells were incubated at 37 °C in a humidified incubator with 5% CO_2_ and tested without mycoplasma contamination.

### Lentivirus production and generation of stable cell lines

The short hairpin RNA (shRNA) constructs targeting SMYD3 and the negative control shRNA construct were purchased from Shanghai GeneChem Co., Ltd. The sequences targeting SMYD3 were: shSMYD3 #1, 5’-CCTGATTGAAGATTTGATT-3’ and shSMYD3#2, 5’-CAACTCTTTCACCATCTGTAA-3’. The negative control (NC) shRNA sequence was 5’-TTCTCCGAACGTGTCACGT-3’. The recombinant plasmid containing human full cDNA sequence of SMYD3 and the vector plasmid were purchased from Shanghai GeneChem Co., Ltd. Then, the lentiviral plamids and the packaging plasmid psPAX2, and the VSV-G envelope plasmid pMD2.G were cotransfected into 293 T cells to generate recombinant lentivirus according to the manufacturer’s manual. Cells were infected with lentivirus plus 8 mg/mL polybrene (Sigma-Aldrich, Missouri, USA). Stable cell lines were established by selection with puromycin (2 μg/mL; InvivoGen, USA) for two weeks. The SMYD3 WT and SMYD3 ΔEEL constructs were purchased from Shanghai Fanxu Bio Co., Ltd. The cDNAs of SMYD3 WT and SMYD3 ΔEEL were also mutated in the shSMYD3 #1 target sequence and were amplified by PCR and inserted into the pLenti-CMV-RFP-BSD vector. SMYD3-knockdown OCI-LY8 cells (shSMYD3 #1) were infected with lentivirus containing SMYD3 WT and SMYD3 ΔEEL plus 8 mg/mL polybrene respectively, and stable cell lines were selected with blasticidin (10 μg/mL; InvivoGen, USA) for two weeks.

### Cell viability and proliferation assays

The DLBCL cells were seeded on 96-well plates at a density of 2000 cell per well. During a 5-day culture period, living cells were detected by Cell Counting Kit-8 (Dojindo, Kyushu, Japan) every 24 h according to the manufacturer’s manual.

### Measurement of glucose uptake, lactate production, pyruvate level and extracellular acidification rate (ECAR)

The glucose uptake, lactate production and pyruvate level of cell lines were examined by the Glucose Uptake Colorimetric Assay Kit (K676-100), Lactate Colorimetry Assay Kit II (K627-100) and Pyruvate Colorimetric/Fluorometric Assay Kit (K609-100) from BioVision, Inc (CA, USA) according to the manufacturer’s instructions.

The glycolytic capacity was measured using the Seahorse XFp Glycolysis Stress Test Kit and the Bioscience XF96 Extracellular Flux Analyzer according to the manufacturer’s instructions. Briefly, 1 × 10^5^ cells were seeded on 96-well plates that were previously treated with poly-D-lysine (PDL) and then incubated for 1 h. After washing the cells with Seahorse buffer (IMEM with phenol red containing 25 mmol/L glucose, 2 mmol/L sodium pyruvate, and 2 mmol/L glutamine), 25 mL each of 10 mmol/L glucose, 1 mmol/L oligomycin, and 100 mmol/L 2-deoxy-glucosewere was added to measure ECAR.

### In vivo xenograft tumor model

Animal studies were approved by the Ethics Committee of Fudan University Shanghai Cancer Center. Briefly, forty female BALB/c nude mice (Shanghai Slac Laboratory Animal Co., Ltd, 6 weeks) were subcutaneously injected with SMYD3-knockdown OCI-LY1 cells (OCI-LY1-shSMYD3 #1, 1 × 10^7^ suspended in 0.1 mL PBS with 0.1 mL Matrigel for each mouse) and OCI-LY8 cells (OCI-LY8-shSMYD3 #1, 5 × 10^6^ suspended in 0.1 mL PBS with 0.1 mL Matrigel for each mouse) and their negative control cells (OCI-LY1-NC and OCI-LY8-NC) respectively. Every group had 10 mice and the mice were divided into each group by randomization. Measurement of tumor growth with a digital caliper was performed every 3 days. Once an average tumor volume of 100 mm^3^ was reached, all the mice were subjected to PET/CT scan. The glucose uptake of tumors was evaluated by the standard uptake value (SUV). Finally, the mice were weighed and sacrificed, and the tumors were weighed and dissected. IHC of xenograft tumors was performed according to the protocol above. The animal studies were performed in a blind manner.

### Western blotting

Western blotting was performed on extracted protein samples using anti-SMYD3 (Abcam, ab187149, 1:1000 dilution), and anti-vinculin (Abcam, ab219649, 1:1000 dilution) antibodies as well as anti-PKM2 antibody (Cell Signaling Technology, #4053, 1:3000 dilution). Vinculin was used as the loading control. Briefly, harvested cells were washed with cold PBS and then lysed in RIPA lysis buffer (Thermo Fisher Scientific, Inc.). Protein was separated by SDS-PAGE and then transferred to PVDF membranes (Millipore, MA, USA). The membranes were incubated with antibodies overnight at 4 °C. After incubation, the membranes were washed with TBST (1*TBS containing 0.1% Tween-20), followed by incubation with HRP-conjugated secondary antibody for 1 h at room temperature. Washed again with TBST, the immunoreactive bands were visualized with Bio-rad Image analysis systems (Bio-Rad, Hercules, CA, USA).One loading control was performed for the proteins on the same membrane. Full length western blots were provided in the Supplementary materials.

### Coimmunoprecipitation (Co-IP) and mass spectrometry

Whole cell lysates of two 75 cm flasks with OCI-LY8 cells at 80% confluency were prepared using RIPA lysis buffer (P0013D, Beyotime Biotechnology). Then, co-IP was performed using an anti-SMYD3 antibody, and the immune complexes were purified on protein A/G agarose beads (Thermo Fisher Scientific, Inc.). After five washes with PBS, the immune complexes were dissolved in 1 × SDS buffer and separated in a 10% SDS–PAGE gel and stained with Coomassie blue. The different gel bands and their corresponding negative gel bands were excised and digested using trypsin. The harvested peptides were detected using nano-HPLC-MS/MS analysis by Q Exactive™ Plus coupled to an EASY-nanoLC 1200 system (Thermo Fisher Scientific, Inc).

### RNA-Seq and data analysis

Total RNA isolated from stable SMYD3-knockdown OCI-LY8 cells (shSMYD3 #1 and shSMYD3 #2) and NC cells was used for RNA-Seq to profile differentially expressed mRNA transcripts. RNA-seq libraries were prepared using VAHTS mRNA-seq v2 Library Prep Kit for Illumina (Vazyme Biotech Co., Ltd.) following the manufacturer’s instructions and sequenced using the Illumina sequencing platform (Xten). Fold change > 1.5 or < 0.7 with a *P* < 0.05 was used as the threshold to identify differentially expressed mRNAs in these cells vs. control cells. Gene set enrichment analysis (GSEA) and Ingenuity Pathway Analysis (IPA) were used for the pathway analysis. The RNA-seq data was deposited into the Gene Expression Omnibus (GEO) database (https://www.ncbi.nlm.nih.gov/geo/query/acc.cgi?acc=GSE207262).

### Chromatin immunoprecipitation (ChIP) and ChIP-qPCR

ChIP assays with OCI-LY8 cells were performed using a ChIP assay kit (#9003, Cell Signaling Technology, Inc.) according to the manufacturer’s instructions. Antibodies specific to SMYD3 (ABE2870), H3K4me3 (17–614) and H3 (17–10046) from Sigma-Aldrich (Missouri, USA) were used for immunoprecipitation. Immunoprecipitated DNA was analyzed with primers that amplified the different regions of *PKM2*. The amplified DNAs were subjected to DNA agarose gel electrophoresis and were also analyzed by qPCR. The primers used for ChIP-qPCR are summarized in Table S8. ChIP assay was performed in triplicate and repeated independently three times.

### Statistical analysis

All experiments were performed in triplicate and repeated independently at least three times. The data were expressed as the mean ± standard deviation, and the differences among the groups were analyzed using Mann-Whitney U test. The chi-square test was used to analyze the relationship between SMYD3 expression and clinicopathological characteristics. The association between the expression levels of two genes was determined using Spearman’s correlation analysis. Kaplan-Meier curves, the log-rank test and Cox univariate and multivariate analysis were performed to analyze patient survival stratified by gene expression. Univariate and multivariate logistic regression were used to analyze the association of SMYD3 expression with the chemotherapy responsiveness. A power analysis indicated that the tissue sample size of 217 and animal sample size of 40 had adequate power based on the results from Zhang et al. [47]. A two-sided *P*-value < 0.05 was considered statistically significant. Statistical analyses were performed using SPSS 20.0 (IBM Corp.) or GraphPad Prism 5.0 (GraphPad Software, Inc.). R software version 4.1.2 (https://www.r-project.org/) was used to analyze GEO dataset.

## Supplementary information

Supplementary information

Supplementary Table 1

Supplementary Table 2

Supplementary Table 3

Supplementary Table 4

Supplementary Table 5

Supplementary Table 6

Supplementary Table 7

Supplementary Table 8

Supplementary Figure 1

Supplementary Figure 2

Supplementary Figure 3

Supplementary Figure 4

Supplementary Figure 5

full western blots

reproducibility checklist

## Data Availability

The datasets used and/or analyzed during the current study are available from the corresponding author on reasonable request.
