# Supplementary material for: SMYD3 promotes aerobic glycolysis in diffuse large B-cell lymphoma via H3K4me3-mediated PKM2 transcription
Source: Cell Death Dis. 2022 Sep 3;13(9):763. doi: 10.1038/s41419-022-05208-7 (PMC9440895; doi:10.1038/s41419-022-05208-7)
Supplement: Supplementary file 1 — Supplementary information [file 41419_2022_5208_MOESM1_ESM.docx]

**Supplementary Figure legends**

**Supplementary Figure 1 The expression of HMT genes and its association with PFS and OS in DLBCLs in the public database.** The lower mRNA expression of EHMT1, NSD1, NSD3 and SETD1A was significantly associated with poor PFS (A) and OS (B) in DLBCL patients based on the GSE87371 dataset; (C) The mRNA levels of EHMT1, NSD1, NSD3 and SETD1A were significantly higher than those of normal tissues in the TCGA database.

**Supplementary Figure 2 Overexpression of SMYD3 promoted DLBCL cell proliferation in HBL1 cell line.** (A, B) Stable overexpression of SMYD3 was measured in HBL1 cell by qRT-PCR and western blot. (C) Overexpression of SMYD3 promoted cell proliferation in HBL1 cell line.

**Supplementary Figure 3 GSEA analysis of the RNA-seq results in SMYD3-knockdown cells vs. NC cells.** (A) A Venn diagram depicting 2267 overlapping differentially expressed genes (DEGs) in OCI-LY8 cells infected with two shRNAs against SMYD3 (shSMYD3 #1 and shSMYD3 #2) vs. NC-treated LY8 cells shown in the interaction of the circles. The left circle included 3991 significant DEGs of shSMYD3 #1 vs NC and the right circle included 3229 significant DEGs of shSMYD3 #2 vs NC. The heatmap showed the hierarchical clustering analysis of the overlapping DEGs (bottom). GSEA of KEGG analysis showed that ‘Glycolysis-Gluconeogenesis’ pathway was upregulated in phenotype shSMYD3 #1 (B) and shSMYD3 #2 (C). The Y-axis plotted the enrichment score and X-axis was the rank of the DEGs. Bar codes below enrichment plots showed the rank position of individual DEGs.

**Supplementary Figure 4 The protein expression of PKM1 and PKM2 in DLBCL.** Immunohistochemistry assay showed that the staining intensity and proportion of PKM1 was very low (H-score: 0, A), while the PKM2 staining intensity was moderate and the staining proportion of PKM2 was about 70% (H-score: 140, B) Magnification,x200. Scale bar, 100μm

**Supplementary Figure 5 SMYD3 induced the resistance of vincristine in DLBCL cells.** IC50 values for vincristine in OCI-LY1 (A) and OCI-LY8 (B) cell lines were obtained using Cell Counting Kit-8 assays. SMYD3 knockdown cells proliferated much more slowly after treatment with vincristine over a period of 5 days in OCI-LY1 (C) and OCI-LY8 (D) cells.

**Supplementary Table legends**

**Supplementary Table 1** HMT genes analyzed in our study

**Supplementary Table 2** Differentially expressed statistics of LC-MS data

**Supplementary Table 3** Overlapping pathways between LC-MS and RNA-seq

**Supplementary Table 4** Associations of PKM2 protein expression with the clinicopathologic characteristics of DLBCLs

**Supplementary Table 5** Univariate and multivariate analysis for associations of PKM2 protein expression with OS in DLBCLs

**Supplementary Table 6** Univariate and multivariate analysis for associations of SMYD3 protein expression with the chemotherapy response in DLBCLs

**Supplementary Table 7** RT-qPCR primers

**Supplementary Table 8** ChIP-qPCR primers

**Full length western blots**
