# Supplementary material for: SMYD3 promotes aerobic glycolysis in diffuse large B-cell lymphoma via H3K4me3-mediated PKM2 transcription
Source: Cell Death Dis. 2022 Sep 3;13(9):763. doi: 10.1038/s41419-022-05208-7 (PMC9440895; doi:10.1038/s41419-022-05208-7)
Supplement: Supplementary file 2 — Supplementary Table 1 [file 41419_2022_5208_MOESM2_ESM.docx]

| **Supplementary Table 1 HMT genes analyzed in our study** | | | |
| --- | --- | --- | --- |
| HMT family | Gene name | Target histones | Related tumors |
| ASH1L | ASH1L | H3K36 | Acute myeloid leukemia (1) |
| DOT1L | DOT1L | H3K79 | Lung cancer (2), breast cancer (3) |
| EHMT | EHMT1, EHMT2 | H3K9 | Breast cancer (4) |
| EZH2 | EZH2 | H3K27 | Prostate cancer, breast cancer, DLBCL (5, 6) |
| NSD | NSD1(KMT3B), NSD2(WHSC1/MMSET) NSD3 (WHSC1L1) | H3K36 | Breast cancer (7) |
| SETD | SETD7, SETD8, SETD1A, SETDB2, SETD7, SETD8 | H3K4 | Breast cancer, prostate cancer, gastric cancer (8) |
| SMYD | SMYD2, SMYD3, SMYD4, SMYD5 | H3K4, H3K36 | Breast cancer, lung cancer, colorectal cancer et al (9) |
| SUV | SUV39H1, SUV39H2, SUV420H1 | H3K9, H4K20 | Cervical cancer, hepatocellular carcinoma, breast cancer (10) |
| KMT | KMT2A, KMT2D | H3K4 | DLBCL (11), prostate cancer (12) |

Reference

1. Rogawski DS, Deng J, Li H, Miao H, Borkin D, Purohit T, et al. Discovery of first-in-class inhibitors of ASH1L histone methyltransferase with anti-leukemic activity. Nature communications. 2021;12(1):2792.

2. Marsh DJ, Ma Y, Dickson KA. Histone Monoubiquitination in Chromatin Remodelling: Focus on the Histone H2B Interactome and Cancer. Cancers (Basel). 2020;12(11).

3. Duan Y, Zhang X, Yang L, Dong X, Zheng Z, Cheng Y, et al. Disruptor of telomeric silencing 1-like (DOT1L) is involved in breast cancer metastasis via transcriptional regulation of MALAT1 and ZEB2. Journal of genetics and genomics = Yi chuan xue bao. 2019;46(12):591-4.

4. Curry E, Green I, Chapman-Rothe N, Shamsaei E, Kandil S, Cherblanc FL, et al. Dual EZH2 and EHMT2 histone methyltransferase inhibition increases biological efficacy in breast cancer cells. Clinical epigenetics. 2015;7(1):84.

5. Park SH, Fong KW, Mong E, Martin MC, Schiltz GE, Yu J. Going beyond Polycomb: EZH2 functions in prostate cancer. Oncogene. 2021;40(39):5788-98.

6. Adibfar S, Elveny M, Kashikova HS, Mikhailova MV, Farhangnia P, Vakili-Samiani S, et al. The molecular mechanisms and therapeutic potential of EZH2 in breast cancer. Life sciences. 2021:120047.

7. Chang S, Yim S, Park H. The cancer driver genes IDH1/2, JARID1C/ KDM5C, and UTX/ KDM6A: crosstalk between histone demethylation and hypoxic reprogramming in cancer metabolism. Experimental & molecular medicine. 2019;51(6):1-17.

8. Singh PK. Histone methyl transferases: A class of epigenetic opportunities to counter uncontrolled cell proliferation. European journal of medicinal chemistry. 2019;166:351-68.

9. Bernard BJ, Nigam N, Burkitt K, Saloura V. SMYD3: a regulator of epigenetic and signaling pathways in cancer. Clinical epigenetics. 2021;13(1):45.

10. Saha N, Muntean AG. Insight into the multi-faceted role of the SUV family of H3K9 methyltransferases in carcinogenesis and cancer progression. Biochimica et biophysica acta Reviews on cancer. 2021;1875(1):188498.

11. Pasqualucci L, Dalla-Favera R. Genetics of diffuse large B-cell lymphoma. Blood. 2018;131(21):2307-19.

12. Lv S, Ji L, Chen B, Liu S, Lei C, Liu X, et al. Histone methyltransferase KMT2D sustains prostate carcinogenesis and metastasis via epigenetically activating LIFR and KLF4. Oncogene. 2018;37(10):1354-68.
