# Supplementary material for: SMYD3 promotes aerobic glycolysis in diffuse large B-cell lymphoma via H3K4me3-mediated PKM2 transcription
Source: Cell Death Dis. 2022 Sep 3;13(9):763. doi: 10.1038/s41419-022-05208-7 (PMC9440895; doi:10.1038/s41419-022-05208-7)
Supplement: Supplementary file 5 — Supplementary Table 4 [file 41419_2022_5208_MOESM5_ESM.docx]

**Supplementary Table 4 Associations of PKM2 protein expression with the clinicopathologic characteristics of DLBCLs**

| Variables | Total case | Low PKM2  expression  (%) | HighPKM2  expression  (%) | *P*-value |
| --- | --- | --- | --- | --- |
| Age | | | | |
| ≤60 | 104 | 50(48.1) | 54(51.9) | 0.213 |
| >60 | 62 | 36(58.1) | 26(41.9) |  |
| Sex | | | | |
| Male | 95 | 53(55.8) | 42(44.2) | 0.235 |
| Female | 71 | 33(46.5) | 38(53.5) |  |
| Primary site | | | | |
| Nodal | 119 | 58(48.7) | 61(51.3) | 0.208 |
| Extranodal | 47 | 28(59.6) | 19(40.4) |  |
| Ann Arbor Stage | | | | |
| I-II | 101 | 58(57.4) | 43(42.6) | 0.071 |
| III-IV | 65 | 28(43.1) | 37(56.9) |  |
| B Symptoms | | | | |
| Yes | 82 | 46(56.1) | 36(43.9) | 0.274 |
| No | 84 | 40(47.6) | 44(52.4) |  |
| IPI scores | | | | |
| Low(0-2) | 136 | 71(52.2) | 65(47.8) | 0.827 |
| High(3-5) | 30 | 15(50.0) | 15(50.0) |  |
| Serum LDH | | | | |
| Normal(≤240） | 95 | 51(53.7) | 44(46.3) | 0.576 |
| High(>240) | 71 | 35(49.3) | 36(50.7) |  |
| Type(IHC) | | | | |
| GCB | 81 | 48(59.3) | 33(40.7) | 0.061 |
| Non-GCB | 85 | 38(44.7) | 47(55.3) |  |
| Relapse or die in 2 years | | | | |
| Yes | 50 | 20(40.0） | 30(60.0) | 0.046^*^ |
| No | 116 | 66(56.9) | 50(43.1) |  |
| Abbreviations:DLBCL, diffuse large B-cell lymphoma; GCB, germinal center B cell; IHC, immunohistochemistry; IPI, International Prognostic Index; LDH, lactate dehydrogenase; CR, complete response; PR, partial response; PD, progressive response; SD, stable disease  ^a^Regimens without Rituximab include CHOP, CEOP, CTOP, HyperCVAD in our cohort; regimens with Rituximab include R-CHOP,R-CEOP, R-HyperCVAD  ^*^*P* values are significant at *P*< 0.05. | | | | |
