# Supplementary material for: SMYD3 promotes aerobic glycolysis in diffuse large B-cell lymphoma via H3K4me3-mediated PKM2 transcription
Source: Cell Death Dis. 2022 Sep 3;13(9):763. doi: 10.1038/s41419-022-05208-7 (PMC9440895; doi:10.1038/s41419-022-05208-7)
Supplement: Supplementary file 6 — Supplementary Table 5 [file 41419_2022_5208_MOESM6_ESM.docx]

| **Supplementary Table 5 Univariate and multivariate analysis for associations of PKM2 protein expression with OS in DLBCLs** | | | | | | |
| --- | --- | --- | --- | --- | --- | --- |
| Variables | Univariate analysis | | | Multivariate analysis | | |
|  | HR | 95%CI | *P*-value | HR | 95%CI | *P*-value |
| PKM2 | | | | | | |
| Low | 1[Reference] | | | 1[Reference] | | |
| High | 1.808 | 1.081-3.024 | 0.024^*^ | 2.055 | 1.217-3.471 | 0.007^*^ |
| Age | | | | | | |
| ≤60 | 1[Reference] | | |  | | |
| >60 | 1.007 | 0.600-1.691 | 0.978 |  |  |  |
| Sex | | | | | | |
| Male | 1[Reference] | | |  | | |
| Female | 0.733 | 0.435-1.237 | 0.245 |  |  |  |
| Primary site | | | | | | |
| Nodal | 1[Reference] | | |  |  |  |
| Extranodal | 1.456 | 0.859-2.470 | 0.163 |  |  |  |
| Ann Arbor Stage | | | | | | |
| I-II | 1[Reference] | | | 1[Reference] | | |
| III-IV | 3.157 | 1.885-5.287 | <0.001^*^ | 1.596 | 0.847-3.008 | 0.148 |
| IPI scores | | | | | | |
| Low(0-2) | 1[Reference] | | | 1[Reference] | | |
| High(3-5) | 4.484 | 2.620-7.675 | <0.001^*^ | 3.022 | 1.680-5.437 | 0.0001^*^ |
| B symptoms | | | | | | |
| No | 1[Reference] | | | 1[Reference] | | |
| Yes | 1.993 | 1.187-3.346 | 0.009^*^ | 1.952 | 1.147-3.320 | 0.014^*^ |
| Serum LDH | | | | | | |
| ≤240 | 1[Reference] | | | 1[Reference] | | |
| >240 | 3.043 | 1.815-5.102 | <0.001^*^ | 2.016 | 1.143-3.555 | 0.015^*^ |
| Type(IHC) | | | | | | |
| GCB | 1[Reference] | | |  | | |
| Non-GCB | 0.604 | 0.363-1.003 | 0.051 |  |  |  |
| Abbreviations: DLBCL, diffuse large B-cell lymphoma; GCB, germinal center B cell; IHC, immunohistochemistry; IPI, International Prognostic Index; LDH, lactate dehydrogenase; CI, confidence interval; HR, Hazard’s ratio  ^*^*P* values are significant at *P*< 0.05 | | | | | | |
