# Supplementary material for: SMYD3 promotes aerobic glycolysis in diffuse large B-cell lymphoma via H3K4me3-mediated PKM2 transcription
Source: Cell Death Dis. 2022 Sep 3;13(9):763. doi: 10.1038/s41419-022-05208-7 (PMC9440895; doi:10.1038/s41419-022-05208-7)
Supplement: Supplementary file 7 — Supplementary Table 6 [file 41419_2022_5208_MOESM7_ESM.docx]

| **Supplementary Table 6 Univariate and multivariate analysis for associations of SMYD3 protein expression with the chemotherapy response of DLBCLs** | | | | | | |
| --- | --- | --- | --- | --- | --- | --- |
| Variables | Univariate analysis | | | Multivariate analysis | | |
|  | OR | 95%CI | *P*-value | OR | 95%CI | *P*-value |
| SMYD3 | | | | | | |
| Low | 1[Reference] | | | 1[Reference] | | |
| High | 4.818 | 1.434-16.193 | 0.011^*^ | 4.108 | 1.108-15.221 | 0.035^*^ |
| Age | | | | | | |
| ≤60 | 1[Reference] | | |  | | |
| >60 | 0.918 | 0.305-2.758 | 0.878 |  |  |  |
| Sex | | | | | | |
| Male | 1[Reference] | | |  | | |
| Female | 0.383 | 0.115-1.283 | 0.120 |  |  |  |
| Primary site | | | | | | |
| Nodal | 1[Reference] | | |  |  |  |
| Extranodal | 0.369 | 0.078-1.747 | 0.209 |  |  |  |
| Ann Arbor Stage | | | | | | |
| I-II | 1[Reference] | | | 1[Reference] | | |
| III-IV | 7.320 | 2.153-24.883 | 0.001^*^ | 3.920 | 1.057-14.542 | 0.041^*^ |
| IPI scores | | | | | | |
| Low(0-2) | 1[Reference] | | |  | | |
| High(3-5) | 1.222 | 0.238-6.267 | 0.810 |  |  |  |
| B symptoms | | | | | | |
| No | 1[Reference] | | | 1[Reference] | | |
| Yes | 4.588 | 1.366-15.407 | 0.014^*^ | 3.374 | 0.884-12.878 | 0.075 |
| Serum LDH | | | | | | |
| ≤240 | 1[Reference] | | |  | | |
| >240 | 1.380 | 0.468-4.069 | 0.559 |  |  |  |
| Type(IHC) | | | | | | |
| GCB | 1[Reference] | | |  | | |
| Non-GCB | 0.522 | 0.174-1.563 | 0.245 |  |  |  |
| Chemotherapy | | | | | | |
| Without R | 1[Reference] | | |  |  |  |
| With R | 0.547 | 0.183-1.637 | 0.281 |  |  |  |
| Abbreviations: DLBCL, diffuse large B-cell lymphoma; GCB, germinal center B cell; IHC, immunohistochemistry; IPI, International Prognostic Index; LDH, lactate dehydrogenase; CI, confidence interval; HR, Hazard’s ratio  ^*^*P* values are significant at *P*< 0.05 | | | | | | |
