# Supplementary material for: SMYD3 promotes aerobic glycolysis in diffuse large B-cell lymphoma via H3K4me3-mediated PKM2 transcription
Source: Cell Death Dis. 2022 Sep 3;13(9):763. doi: 10.1038/s41419-022-05208-7 (PMC9440895; doi:10.1038/s41419-022-05208-7)
Supplement: Supplementary file 8 — Supplementary Table 7 [file 41419_2022_5208_MOESM8_ESM.docx]

| **Supplementary Table 7 RT-qPCR primers** | |
| --- | --- |
| GENE | Sequence (5’-3’) |
| ACTB-Forward | AGTCATTCCAAATATGAGATGCGTT |
| ACTB-Reverse | TGCTATCACCTCCCCTGTGT |
| SMYD3-Forward | GGGCCCACCTCTTACTGCGA |
| SMYD3-Reverse | CTGCTTCCGCGCTCCTCAC |
| PKM1-Forward | AGAACTTGTGCGAGCCTCAA |
| PKM1-Reverse | GACGAGCTGTCTGGGGATTC |
| PKM2-Forward | GTGATGTGGCCAATGCAGTC |
| PKM2-Reverse | CAAGTGGTAGATGGCAGCCT |
