# Supplementary material for: SMYD3 promotes aerobic glycolysis in diffuse large B-cell lymphoma via H3K4me3-mediated PKM2 transcription
Source: Cell Death Dis. 2022 Sep 3;13(9):763. doi: 10.1038/s41419-022-05208-7 (PMC9440895; doi:10.1038/s41419-022-05208-7)
Supplement: Supplementary file 9 — Supplementary Table 8 [file 41419_2022_5208_MOESM9_ESM.docx]

| **Supplementary Table 8 ChIP-qPCR primers** | | |
| --- | --- | --- |
| GENE | Sequence (5’-3’) |  |
| PKM-A-Forward | TTCACTGAACGCAAACGGTG |  |
| PKM-A-Reverse | CTGGTGAGACCTTACGAGGC |  |
| PKM-B-Forward | TGTTCCGTGCTCGTTTTCCC |  |
| PKM-B-Reverse | GAACGCAGTTTCAATGAGTTACAC |  |
| PKM-C-Forward | GTTCCGCGGTCCTAACACA |  |
| PKM-C-Reverse | GAGCTGAACGGAAAGTTGGG |  |
| PKM-D-Forward | AGGGAGGGACTTTTATGCCA |  |
| PKM-D-Reverse | TGTTGTTGGGAAGGAAATGCTTG |  |
