# Supplementary figures and images for: SMYD3 promotes aerobic glycolysis in diffuse large B-cell lymphoma via H3K4me3-mediated PKM2 transcription
Source: Cell Death Dis. 2022 Sep 3;13(9):763. doi: 10.1038/s41419-022-05208-7 (PMC9440895; doi:10.1038/s41419-022-05208-7)

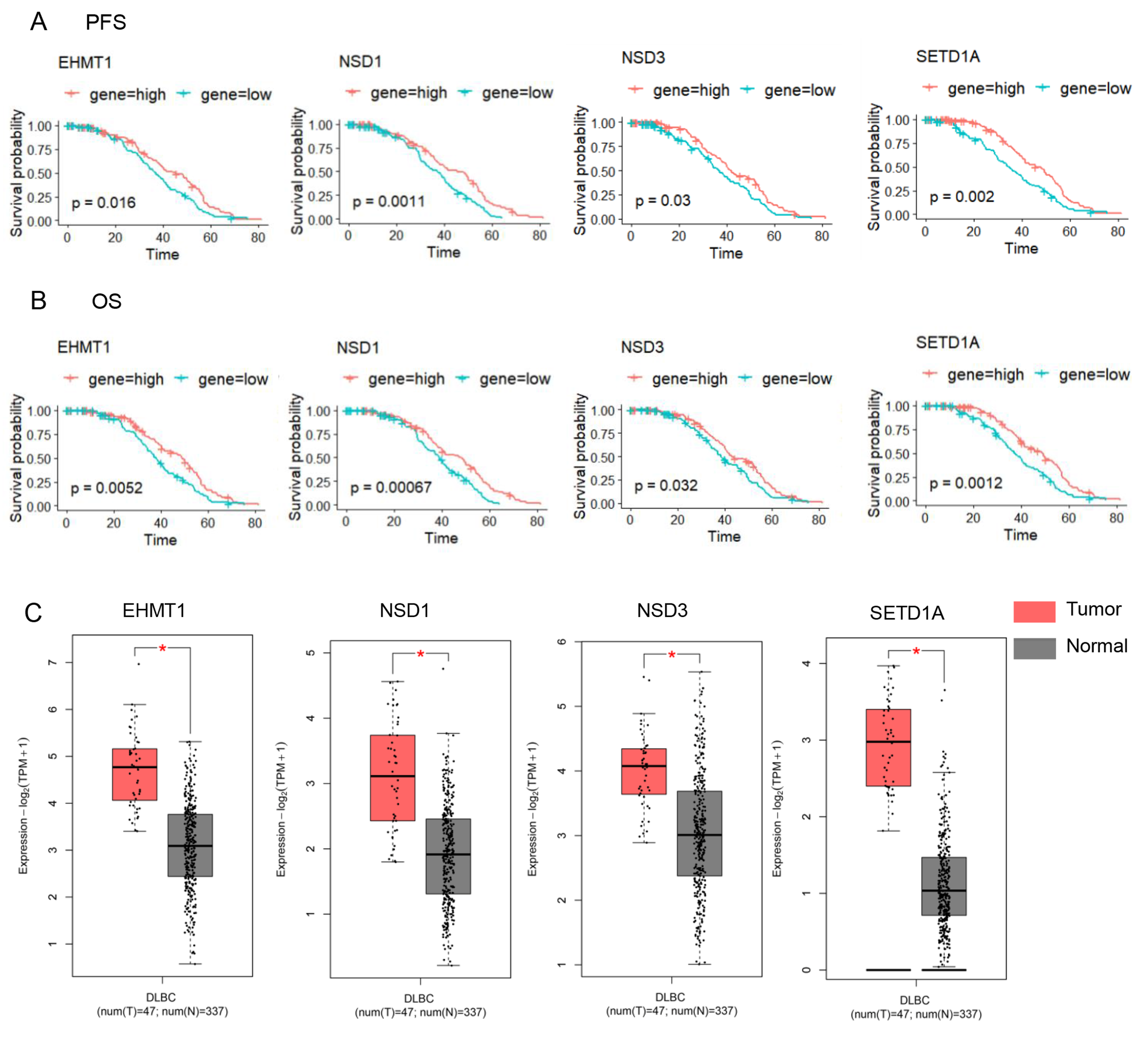

Supplement: Supplementary file 10 — Supplementary Figure 1 [file 41419_2022_5208_MOESM10_ESM.tif]

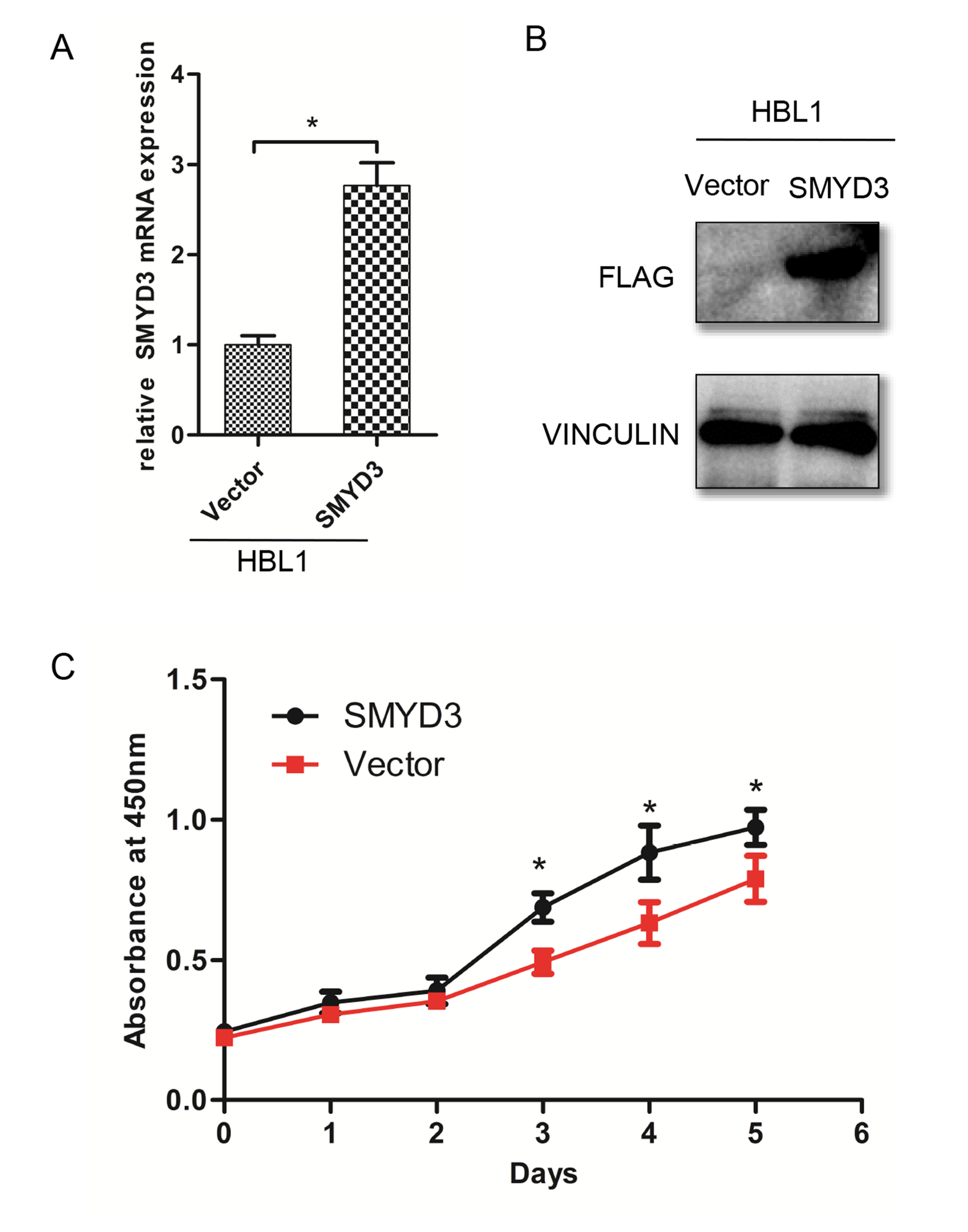

Supplement: Supplementary file 11 — Supplementary Figure 2 [file 41419_2022_5208_MOESM11_ESM.tif]

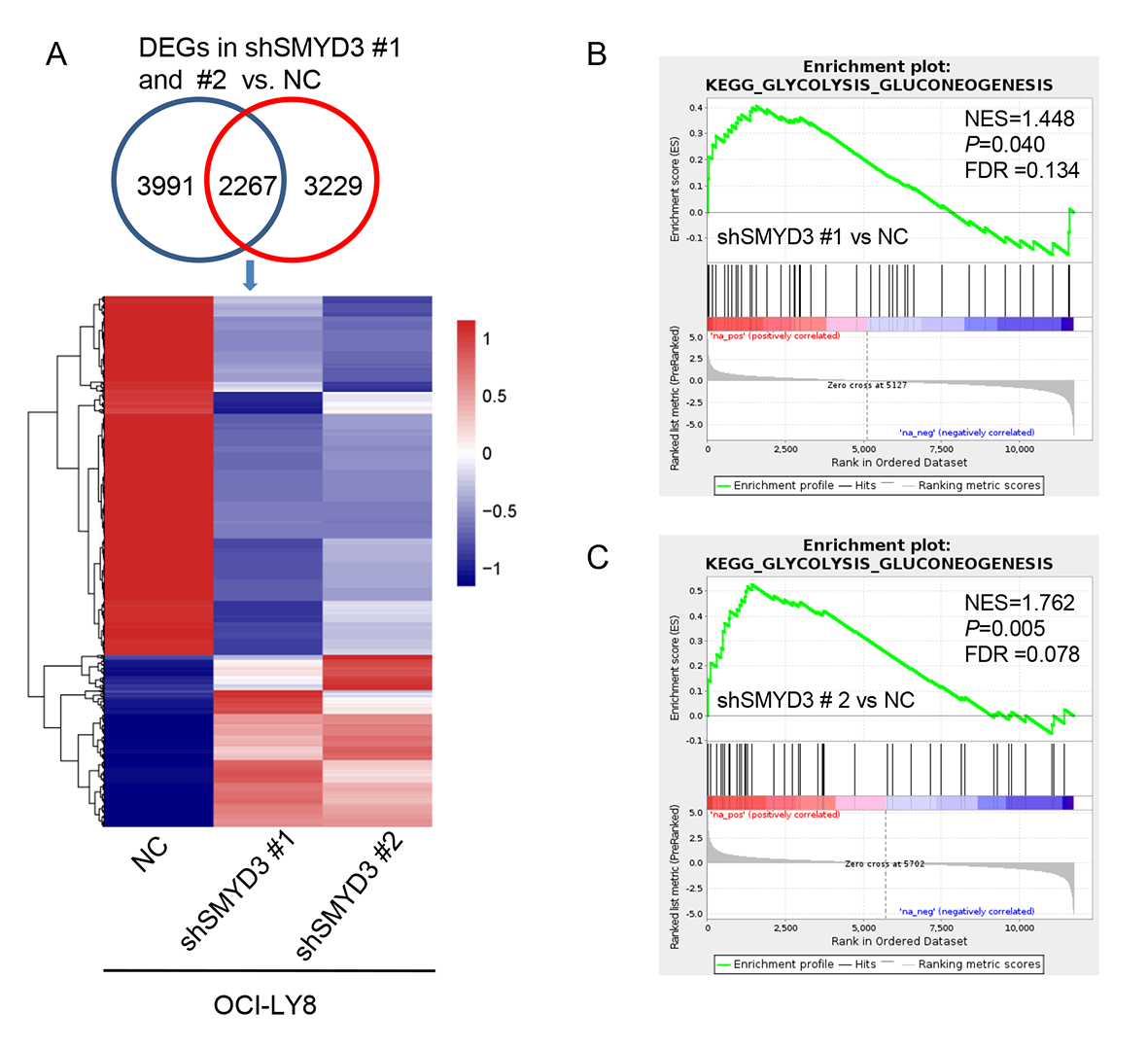

Supplement: Supplementary file 12 — Supplementary Figure 3 [file 41419_2022_5208_MOESM12_ESM.tif]

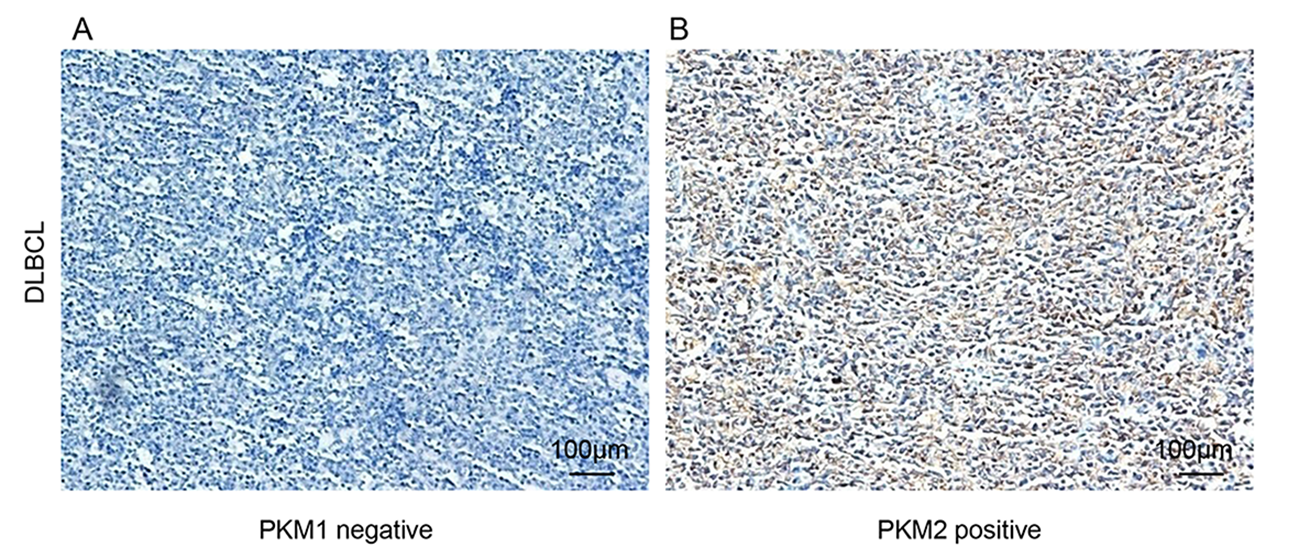

Supplement: Supplementary file 13 — Supplementary Figure 4 [file 41419_2022_5208_MOESM13_ESM.tif]

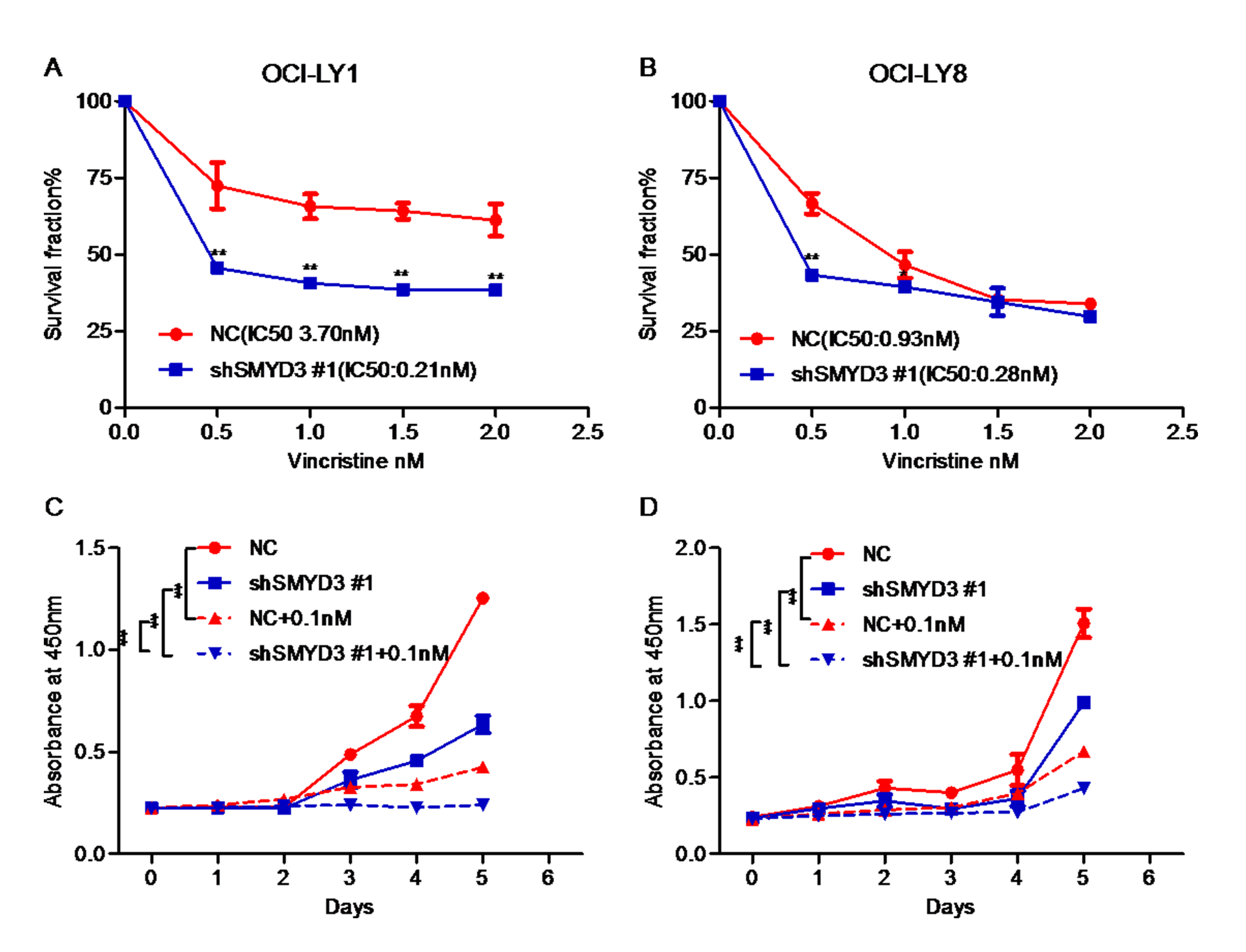

Supplement: Supplementary file 14 — Supplementary Figure 5 [file 41419_2022_5208_MOESM14_ESM.tif]

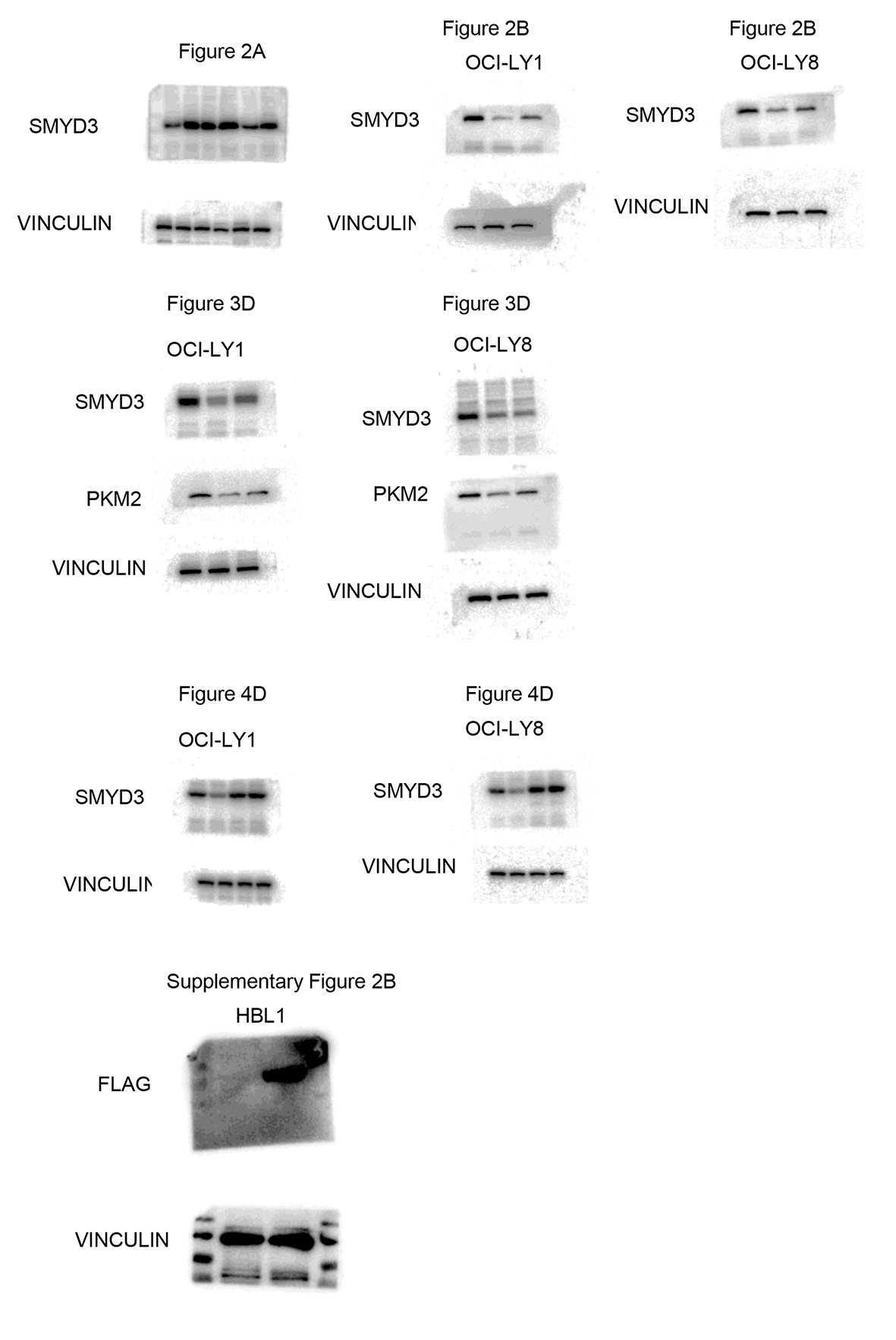

Supplement: Supplementary file 15 — full western blots [file 41419_2022_5208_MOESM15_ESM.tif]
